# A phylogenetic method to perform genome-wide association studies in microbes that accounts for population structure and recombination

**DOI:** 10.1371/journal.pcbi.1005958

**Published:** 2018-02-05

**Authors:** Caitlin Collins, Xavier Didelot

**Affiliations:** Department of Infectious Disease Epidemiology, Imperial College London, London, United Kingdom; Helmholtz-Zentrum fur Infektionsforschung GmbH, GERMANY

## Abstract

Genome-Wide Association Studies (GWAS) in microbial organisms have the potential to vastly improve the way we understand, manage, and treat infectious diseases. Yet, microbial GWAS methods established thus far remain insufficiently able to capitalise on the growing wealth of bacterial and viral genetic sequence data. Facing clonal population structure and homologous recombination, existing GWAS methods struggle to achieve both the precision necessary to reject spurious findings and the power required to detect associations in microbes. In this paper, we introduce a novel phylogenetic approach that has been tailor-made for microbial GWAS, which is applicable to organisms ranging from purely clonal to frequently recombining, and to both binary and continuous phenotypes. Our approach is robust to the confounding effects of both population structure and recombination, while maintaining high statistical power to detect associations. Thorough testing via application to simulated data provides strong support for the power and specificity of our approach and demonstrates the advantages offered over alternative cluster-based and dimension-reduction methods. Two applications to *Neisseria meningitidis* illustrate the versatility and potential of our method, confirming previously-identified penicillin resistance loci and resulting in the identification of both well-characterised and novel drivers of invasive disease. Our method is implemented as an open-source R package called treeWAS which is freely available at https://github.com/caitiecollins/treeWAS.

## Author summary

Measurable differences often exist within a microbial population, with important ecological or epidemiological consequences. Examples include differences in growth rates, host range, transmissibility, antimicrobial resistance, virulence, etc. Understanding the genetic factors involved in these phenotypic properties is a crucial aim in microbial genomics. A fundamental approach for doing so is to perform a Genome-Wide Association Study (GWAS), where genomes are compared to search for genetic markers systematically correlated with the property of interest. If this strategy were implemented naively in microbes, it could lead to spurious results due to the confounding effects of population structure and recombination. Here we present treeWAS, a new phylogenetic method to perform microbial GWAS that avoids these pitfalls. We show, using simulated datasets, that treeWAS is able to distinguish between genetic markers that are truly associated with the property of interest and those that are not. Furthermore, we demonstrate that treeWAS offers advantages in both sensitivity and specificity over alternative cluster-based and dimension-reduction techniques. We also showcase treeWAS in two applications to real datasets from *N. meningitidis*. We have developed an easy-to-use implementation of treeWAS in the R environment, which should be useful to a wide range of researchers in microbial genomics.

This is a PLoS Computational Biology Methods paper.

## Introduction

Owing to rapid progress in sequencing technologies, the accumulation of microbial genome sequences has begun to outpace the development of statistical and computational tools for their analysis. As a result, opportunities to reduce the global burden of infectious disease are missed. Meanwhile, infectious diseases remain accountable for 15% of worldwide annual mortality [1]. Moreover, as globalisation continues to increase the rate and scope of human interaction, with each other and with animals, this process will likely be accompanied by parallel change in the spread and evolution of infectious pathogens [2–5]. Discovering the genetic basis of microbial traits would offer key insights into the biological mechanisms underlying infectious diseases, and would improve our ability to develop drugs and vaccines, target treatments, build predictive tools, benefit from surveillance, and enhance public health.

Genome-wide association studies (GWAS) can be used to make these inferences, linking genotype to phenotype by testing for statistical associations between the two. GWAS have become a tool of choice in human genetics, since the publication of the first such studies in the early 2000s [6–9], leading to the identification of over 11,000 trait-associated single nucleotide polymorphisms (SNPs) in humans [10]. It has been anticipated that by applying GWAS methods to microbes, similar discoveries could be made [11]. Indeed, although the advent of GWAS in microbes has been relatively recent, promising results can already be seen in the literature to date [12–14]. By contrast to GWAS in humans, however, microbial association mapping remains a technical challenge in search of an optimal methodological approach.

The purpose of GWAS is to identify statistically significant associations that may indicate the presence of a causal relationship between genotype and phenotype while rejecting spurious associations arising from confounding factors. In microbes, smaller genome sizes and the ability to manipulate these genomes in the laboratory may improve the power and computational ease of GWAS and facilitate the confirmation of candidate loci [11]. On the other hand, microbial association studies must overcome a multiplicity of confounding factors, such as, the stronger population structure that results from clonal reproduction [15], widespread linkage disequilibrium interrupted unpredictably by homologous recombination [16], diversity in genetic content [17], and variability in the phenotypic probability distribution for a given genotype [18].

Most microbial GWAS analyses to date have made an effort to control for the confounding potential of population structure. The strength of this confounding effect increases both with the degree to which allele frequencies differ between subpopulations in a sample and the extent to which phenotypic states cluster within these lineages or clades [14, 19]. Cluster-based methods [20] and dimension reduction techniques [21, 22] have been adopted to account for population structure in microbial association studies [13, 22–26], and recent refinements of these methods have been proposed to increase their statistical power [27, 28]. Nevertheless, like methods that rearrange the phenotype to assess significance [24, 26, 29], these approaches cannot appropriately evaluate the probability that population substructure will give rise to spurious associations because they do not factor the degree of phenotypic clustering into the analysis. Pairwise methods account for fine scale genetic differences and phenotypic clustering, but discard large volumes of valuable data [29, 30]. Clonal relatedness evidently remains a challenge for microbial GWAS based on these strategies.

Fortunately, clonality also enables the adoption of a phylogenetic solution [31–33]. Phylogenetic trees allow for the detailed identification of genetic relationships, not only at the level of population clusters, but also at the resolution of subpopulations and individual relationships. Adopting a phylogenetic approach does not require evolution to be treated as purely clonal, nor that recombination must be ignored, since the effect of recombination events can be considered within a phylogenetic framework [15, 34]. Nor do they require any loss of information, provided pairwise techniques are not used. Phylogenetic approaches are by far the most popular method to describe microbial population structure, and therefore they are a natural option to control for population structure when performing GWAS in microbes.

Here we propose a new phylogenetic approach to GWAS called treeWAS that is able to overcome many of the limitations of existing microbial GWAS approaches. Within our analytical pipeline, data simulation based on parameters of the empirical dataset under analysis allows us to account for the composition of the genetic dataset, the population structure of the sample, and the confounding effects of recombination. We apply multiple complementary scores of association to enhance statistical power and improve detection of associations underlying subtle and complex phenotypes, such as host association or invasiveness. Below, we present the results of rigorous testing on simulated datasets, and compare performance with alternative approaches, including cluster-based and dimension-reduction methods. We also demonstrate how treeWAS responds to varying levels of recombination and contrast this to previous methods. We show that treeWAS provides both specificity and power in a wide range of settings, and consistently offers the best overall performance. Finally, we present two applications to real data from *Neisseria meningitidis*. First, we investigate penicillin resistance and demonstrate that our approach can confirm known resistance loci. Second, we examine invasive disease, which reveals both previously characterised and novel invasiveness factors and illustrates the ability of our methodology to identify associations when applied to complex phenotypes.

## Materials and methods

### Overview of the treeWAS method

Our central aim is to delineate true signals of association from a noisy background of spurious associations. To accomplish this, our method uses the simulation of a null genetic dataset to establish whether high association score values in the empirical dataset under analysis are likely to be truly significant or may, in fact, arise by chance as a result of confounding factors found in the empirical dataset. Outside of the GWAS literature, similar approaches comparing null and empirical distributions have been used to determine whether inferred ancestry supports the functional linkage of genes [35, 36]. In treeWAS, we characterise the evolutionary parameters of the empirical dataset and use these to generate the simulated genetic dataset, which represents the null hypothesis of no association. This null dataset resembles the empirical dataset in both genetic composition and population structure, but does not have any true association with the phenotype. By comparing associations in the empirical and simulated datasets, we are able to determine which signals of association have sufficient statistical and evolutionary support. This approach makes use of all information contained in the dataset, as well as that inferred in phylogenetic and ancestral state reconstructions. We aim to maintain strict control over the number of false positive findings. This makes possible the application of multiple complementary tests of association, which increases the power to detect associations. The entire treeWAS pipeline is typically completed in a matter of minutes or seconds, depending on dataset size (see S1 Appendix).

### Implementation

The treeWAS approach is implemented in the following steps:

1. **Phylogenetic reconstruction** can be performed within treeWAS by distance-based [37–40] or maximum-likelihood (ML) [41] methods. However, where recombination is expected to distort the clonal genealogy, it is recommended that users provide a tree previously reconstructed by a recombination-aware approach [34, 42, 43]. Tools are provided for integration with ClonalFrameML [42].
2. **Computation of the homoplasy distribution**, containing site-specific numbers of substitutions drawn from the empirical dataset, is performed with the Fitch parsimony algorithm [44].
3. **Simulation of null genetic data** enables the delineation of true associations from spurious associations. We compare the relationships between genotype and phenotype at all loci in the real data to those in a simulated dataset that embodies only potentially confounding factors. Simulation of this “null” genetic dataset is guided by three parameters: (i) the phylogenetic tree, (ii) the homoplasy distribution, (iii) the number of loci to be simulated, *N*_*sim*_, which is recommended to be at least ten times the number of biallelic sites in the empirical dataset. Each of the *N*_*sim*_ loci is simulated along the phylogenetic tree, from root to tips, undergoing a number of substitutions drawn from the homoplasy distribution on branches selected randomly with probabilities proportional to branch length. The original phenotype is maintained across the leaves. By retaining the phylogenetic tree and the distribution of phenotypic states, but reassigning substitutions to new branches, we are able to produce a simulated dataset that resembles the empirical dataset in population structure and genetic composition, including the effects of mutation and recombination. Associations due to confounding factors, but no true associations with the phenotype, are thus recreated by the simulation process.
4. **Ancestral character estimation** is required prior to association testing. A marginal reconstruction of the ancestral states of both genotype and phenotype must be performed via parsimony [45] or ML [46, 47] (see S2 Appendix).
5. **Association testing** is performed by applying three independent tests of association to all loci (see below). Associations between simulated loci and the empirical phenotype are first measured, allowing for the identification of a null distribution of association score statistics under the null hypothesis of no association. Associations between empirical loci and the phenotype are then measured and evaluated with reference to this null distribution.
6. **Identification of significance threshold and associations** proceeds by drawing a threshold in the upper tail of the null distribution, at the value corresponding to a base p-value (e.g., *p* = 0.01) that has been corrected for multiple testing to account for both the number of genetic loci and the three association tests (via Bonferroni correction by default, though False Discovery Rate is also implemented). Among the set of empirical association scores, all values that exceed this threshold are deemed to be statistically significant associations and, thus, candidates for true biological association, pending subsequent confirmatory analyses.

### Tests of association

**Fig 1:**
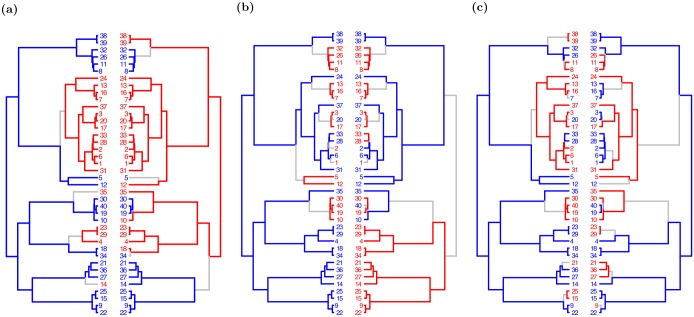
Evolutionary scenarios detected by treeWAS scores. The three complementary tests of association in treeWAS assign high scores to different patterns of association, examples of which are illustrated above. Each panel displays the phenotype (left) and the genotype of one associated locus (right), with binary states plotted along the tips of the phylogenetic tree (N = 40) and reconstructed ancestral states indicated along the branches of the tree (blue = 0, red = 1, grey = substitution). **A:** Score 1 aims to detect association among terminal nodes and assigns a relatively high value of 0.7 to this terminal configuration of phenotypic and genotypic states. **B:** Score 2 measures association by counting how many branches contain a substitution in both genotype and phenotype, assigning this pattern a score of 5. **C:** Score 3 is designed to find associations maintained loosely across the phylogenetic tree, resulting in a Score 3 value of 10 to this scenario.

The design of treeWAS, particularly its use of the null distribution, enables strict control over the false positive rate. This presents the opportunity for power to be augmented by applying multiple independent tests of association. We therefore measure the association between each genetic locus and the phenotype with three separate tests, described below and illustrated in Fig 1. All three tests are applicable to any form of binary genetic data, including SNPs, indels, and gene presence or absence matrices, and can be used on binary phenotypes, discrete interval variables, and continuous phenotypic data. The following notation is used to describe the three association scores. $p_{i}^{anc}$ and $p_{i}^{des}$ denote the phenotypic state at ancestral and descendant node of branch *i*, respectively. $g_{i}^{anc}$ and $g_{i}^{des}$ denote respectively the genotypic state at the ancestral and descendant node of branch *i*. *n* and *n*_*b*_ denote respectively the number of leaves and branches on the phylogenetic tree.

**Score 1**, the “Terminal Score”, measures sample-wide association across the leaves of the phylogenetic tree. For a binary phenotype, this score is equivalent to counting the four terminal state combinations, with and without the phenotype, and with and without the genotype, as previously proposed [48]. Generalizing to continuous phenotypes gives:

$$\begin{array}{c} \text{Score}\;1=|\sum_{i=1}^{n}\frac{1}{n}\left(p_{i}^{des}g_{i}^{des}+\left(1-p_{i}^{des}\right)\left(1-g_{i}^{des}\right)-\left(1-p_{i}^{des}\right)g_{i}^{des}-p_{i}^{des}\left(1-g_{i}^{des}\right)\right)\;| \end{array}$$ (1)

Eq 1 determines whether a given allele is over-represented among individuals of a particular phenotypic state. This allows Score 1 to detect associations that are upheld across a relatively large proportion of terminal nodes. Uniform association in all but six of the terminal nodes in Fig 1A, for example, leads to a high Score 1 value. In addition, because Score 1 is blind to ancestral information, it can detect terminal association even in the absence of strong ancestral indicators of association. Furthermore, its inferences remain robust to any incorrect estimates of the phylogenetic tree or ancestral state reconstructions.

**Score 2**, the “Simultaneous Score”, measures the degree of parallel change in the phenotype and genotype across branches of the tree. For a binary phenotype with a parsimonious ancestral state reconstruction, as in Fig 1B, this means counting the number of branches containing a simultaneous substitution in genotype and phenotype [31]. A more general definition is given by:

$$\begin{array}{c} \text{Score}\;2=|\sum_{i=1}^{n_{b}}\left(p_{i}^{anc}-p_{i}^{des}\right)\left(g_{i}^{anc}-g_{i}^{des}\right)\;| \end{array}$$ (2)

Unlike Score 1, Score 2 is able to use information contained in the tree structure and set of ancestral character states towards the detection of significant associations. In Fig 1B, for example, Score 2 reveals the presence of strong signals of association by detecting five instances of simultaneous substitution. Moreover, because Eq 2 imparts a cumulative character (simultaneous substitutions increase the score, but branches where one or no variable changes do not decrease it), significance by Score 2 does not require sample-wide association. Score 2 may therefore be able to detect loci giving rise to the phenotype through complementary pathways, in addition to identifying loci whose associations with the phenotype persist across the tree.

**Score 3**, the “Subsequent Score”, measures the proportion of the tree in which the genotype and phenotype co-exist. It is the mathematical solution to the integral of an association score along all points of the phylogenetic tree (see S3 Appendix):

$$\begin{array}{c} \;\text{Score}\;3=|\sum_{i=1}^{n_{b}}\frac{4}{3}p_{i}^{anc}g_{i}^{anc}+\frac{2}{3}p_{i}^{anc}g_{i}^{des}+\frac{2}{3}p_{i}^{des}g_{i}^{anc}+\frac{4}{3}p_{i}^{des}g_{i}^{des}-p_{i}^{anc}-p_{i}^{des}-g_{i}^{anc}-g_{i}^{des}+1| \end{array}$$ (3)

Score 3 considers the maintenance of allelic enrichment in a given phenotypic state, as well as the change in both genotype and phenotype. Should association arise by a substitution in one variable being followed on a subsequent branch by a substitution in the other, as in Fig 1C, Score 3 will incur no penalty for the lack of simultaneous change and will capture the downstream association in so far as it is maintained. In host association, for example, where genetic adaptation may contribute to host switching by increasing affinity for a different host or by offering compensatory fitness advantages once in a new environment, Score 3 may be most effective [48]. Overall, this score should be sensitive to subtler and more probabilistic patterns of association.

**Pooling the scores** allows treeWAS to obtain a comprehensive picture of the association present in a dataset. As Fig 1 illustrates, the three scores complement each other and contribute distinct, if overlapping, patterns of association to the output of treeWAS. Score 1 recognises widespread terminal association in Fig 1A and returns a high score, while a Score 2 of zero and a low Score 3 result from their focus on ancestral co-evolution. By contrast, in Fig 1B, where one major clade and 50% of terminal nodes are out of association, we see a Score 1 of zero and a low Score 3, meanwhile the repeated identification of simultaneous substitutions results in a high Score 2. The lack of terminal association and absence of simultaneous substitutions in Fig 1C cause both Scores 1 and 2 to amount to zero, but relaxing both of their requirements and allowing substitutions to occur on subsequent branches results in a high value for Score 3.

Independently, each association test identifies a set of statistically significant genetic loci, and each of these findings constitutes a suitable candidate for further investigation. Although it may provide further support for a finding, identification by a second or third association test is not required for overall significance. Once identified, the three sets of significant genetic loci are pooled together and returned as the set of findings identified by treeWAS. Instead of merging the three well-defined association scores into an uninformative aggregate score, we report the significant association scores and p-values for each test separately. These measures enhance the interpretability of the output of treeWAS, by providing insight into the nature of the associations detected, alongside the list of significant findings.

### Assessment of performance on simulated data

To evaluate the performance of treeWAS, we applied it and six alternative methods to 400 datasets simulated via three separate approaches. Approaches differed only in the nature of the simulated associations between genotype and phenotype. We present one approach below and the other two in S4 and S5 Appendices. Additional results based on the approach below are presented in S6 Appendix.

Each simulated dataset contained 100 individuals and 10,000 binary loci, of which ten loci were associated with a binary phenotype. Variation in the background recombination rate, the ancestral relationships between genomes, the degree of phenotypic clustering among related isolates, and the effect size of associations added complexity and noise to the simulated data and increased the challenge of association testing for treeWAS and all comparator methods. The non-associated loci were simulated using homoplasy distributions corresponding to four recombination rates (0, 0.01, 0.05, 0.1) (see S7 Appendix). Genetic data was simulated along randomly generated coalescent trees. For each simulated non-associated locus, the number of substitutions was drawn from the homoplasy distribution and assigned to branches of the phylogenetic tree with probabilities proportional to branch lengths. The ten associated loci were generated together with the phenotype according to an instantaneous transition rate matrix, *Q*, which controls the rates of transition between all four possible combinations of a binary genetic locus, *G*, and the binary phenotype, *P*, (i.e., *G*_0_ *P*_0_, *G*_0_ *P*_1_, *G*_1_ *P*_0_, *G*_1_ *P*_1_) between an ancestral node (in the rows) and a descendant node (in the columns):

$$\begin{array}{ll} Q= & \begin{array}{ll} \begin{array}{l} {\text{G}}_{0}{\text{P}}_{0}\\ {\text{G}}_{0}{\text{P}}_{1}\\ {\text{G}}_{1}{\text{P}}_{0}\\ {\text{G}}_{1}{\text{P}}_{1} \end{array} & \begin{array}{l} \begin{array}{llll} {\text{G}}_{0}{\text{P}}_{0} & {\text{G}}_{0}{\text{P}}_{1} & {\text{G}}_{1}{\text{P}}_{0} & {\text{G}}_{1}{\text{P}}_{1} \end{array}\\ \left(\begin{array}{llll} -2s & s & s & 0\\ sa & -2sa & 0 & sa\\ sa & 0 & -2sa & sa\\ 0 & s & s & -2s \end{array}\right) \end{array} \end{array} \end{array}$$ (4)

The *Q* matrix is parameterised by *s*, which controls the baseline substitution rate and applies to all columns, and *a*, an association factor that establishes the preference for one form of association (*G*_0_ *P*_0_, *G*_1_ *P*_1_) over the opposite (*G*_0_ *P*_1_, *G*_1_ *P*_0_). The parameter *s* is divided by the sum of the branch lengths before building *Q*. In all simulations, initial parameters were set to *s* = 20 and *a* = 10. To identify the probabilities of transition for a branch of length *l*, the instantaneous transition rate matrix, *Q*, is converted into a matrix of probabilities, *P* = *exp*(*Ql*), via matrix exponentiation, which takes into account the length *l* of the branch in question.

### Comparison with other GWAS methods

To each of the simulated datasets, in addition to treeWAS, we applied six alternative GWAS methods. The Fisher’s exact test, and the *χ*^2^ test available in PLINK version 1.07 [49] were used as benchmarks to demonstrate what results would be found by two standard tests of association without population structure control. The PLINK *χ*^2^ test with Genomic Control (GC), has been used in bacterial GWAS [13] and provided a simple solution to population structure. Principal Components Analysis (PCA) and Discriminant Analysis of Principal Components (DAPC) represent more advanced and popular approaches to correcting for population structure [21, 22]. PCA is the “gold standard” method used in human GWAS [19, 50] and DAPC has been proposed as a potential improvement on PCA [22]. Both have been used in microbial GWAS [22, 25–28]. We followed the standard protocol used in human genetics and corrected for ancestry by regressing along the significant Principal Components (PCs) of PCA or DAPC (see S8 Appendix), and identified significant associations via *χ*^2^ test [19]. The Cochran-Mantel-Haenszel (CMH, [51]) provided an alternative, cluster-based approach. The CMH test works directly with *K* population clusters by adopting a stratified 2x2x*K* design and has been used in bacterial GWAS [13, 23, 24].

## Results/Discussion

### Assessment of treeWAS performance on simulated data

**Fig 2:**
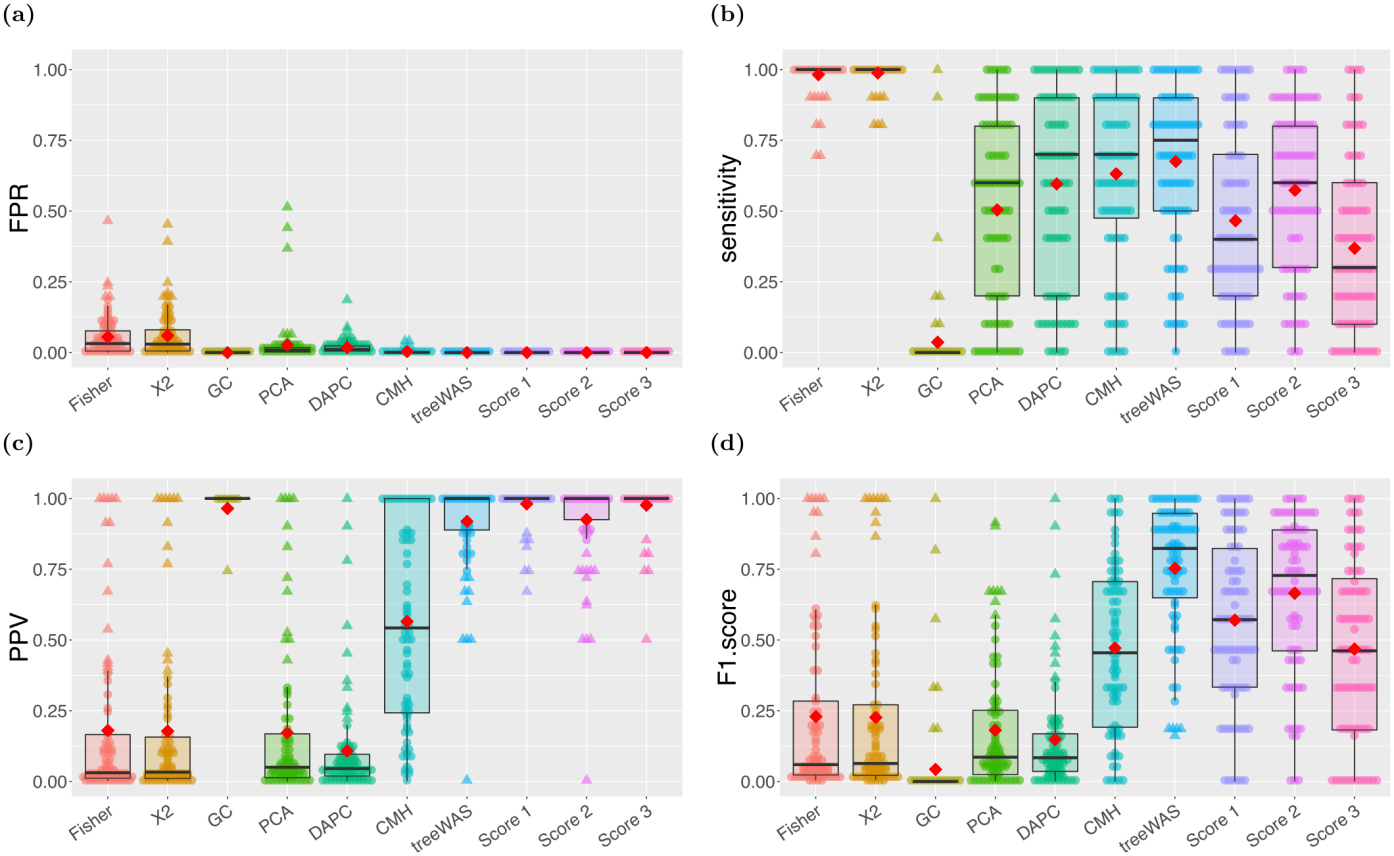
Performance by association test. The performance on simulated datasets for the six comparator GWAS methods and treeWAS, alongside its three association tests individually, is summarised along the four metrics of evaluation. Box plots display the median and interquartile range, red diamonds indicate the mean, and individual dots represent results for one of the 80 simulated datasets. **A:** False Positive Rate. **B:** Sensitivity. **C:** Positive Predictive Value. **D:** F1 Score.

The performance on simulated datasets was evaluated along four metrics: the False Positive Rate (FPR), Sensitivity, Positive Predictive Value (PPV), the proportion of results that are true positives, and the F1 Score, which is the harmonic mean of Sensitivity and PPV [52]. Our approach performed well along all four metrics. Fig 2A shows that treeWAS was able to consistently achieve a FPR of zero, indicating tight control over multiple confounding factors. In Fig 2B, we see that the sensitivity of treeWAS varied between zero and one, taking on 11 different values representing how many of the 10 associated loci were correctly identified. While our conservative approach produces moderate sensitivities in the three individual association scores within treeWAS, the contribution of true positive findings by each score gives treeWAS a very high sensitivity overall. Notably, although Score 2 generally achieved higher sensitivity than Scores 1 and 3, it was not always the leading contributor to the cumulative sensitivity of treeWAS in the analysis of these simulated datasets. This highlights the value of using multiple complementary measures to identify associations. Importantly, cumulative benefits in sensitivity were not undermined by cumulative reductions in PPV. Fig 2C reflects the fact that in most cases the total number of false positives found by treeWAS was zero or one. Overall, the high performance of our approach, as indicated by the composite F1 score in Fig 2D, provides strong support for the strategy adopted by treeWAS. This remains true when the number of individuals or loci simulated is varied (see S9 Appendix).

**Fig 3:**
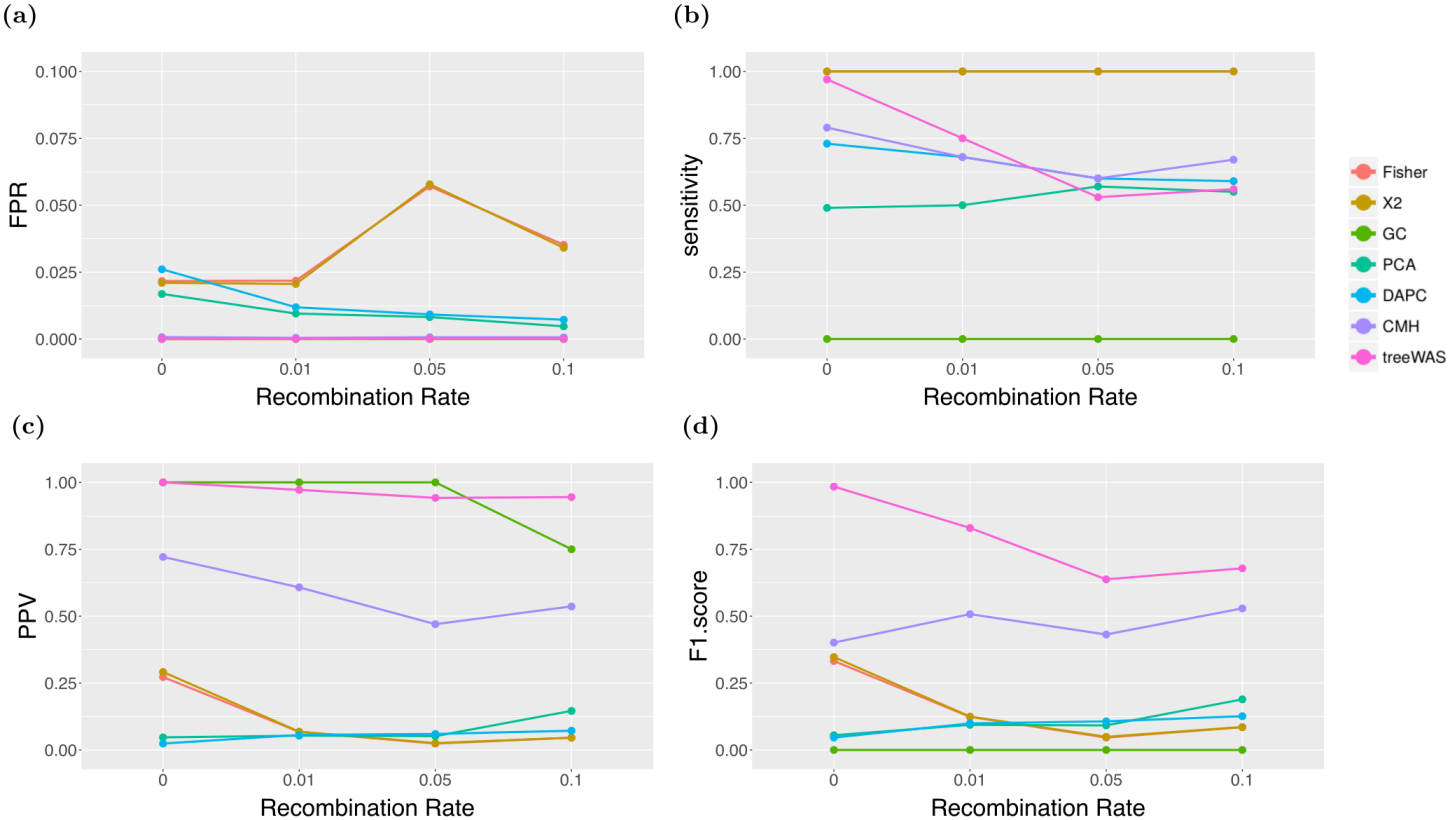
Performance by recombination rate. Interquartile mean performance by GWAS method and recombination rate is plotted along four statistics. **A:** False Positive Rate. **B:** Sensitivity. **C:** Positive Predictive Value. **D:** F1 Score.

In addition to providing a thorough control over population structure, treeWAS was able to account explicitly for the varying confounding effect of recombination. In the simulated datasets analysed above, the phenotype and associated loci underwent between four and 23 binary state substitutions as a result of the probabilistic simulation process, with an average of 14 substitutions per tree. As such, the probability of chance correlation with the resulting pattern of phenotypic clustering increased as the number of substitutions among non-associated loci was elevated to similar levels by recombination (see S7 Appendix). Fig 3A shows that the FPR of treeWAS remained consistently low as the recombination rate was increased. Because data simulation within treeWAS is guided by the empirical homoplasy distribution, the elevated risk of chance association due to recombination was accounted for in the null distribution. Fig 3B illustrates a second implication of this feature: in this analysis, sensitivity decreased with increasing recombination, as treeWAS could no longer attribute significance to some more weakly associated loci when similar patterns of association were likely to occur by chance. Taking into account the parameters of the data causes the impact of recombination on the sensitivity of treeWAS to vary by context (see S4 and S5 Appendices). This data-dependent behaviour is necessary to keep FPR at a minimum (Fig 3A). We do nevertheless see in Fig 3C a slight decline in the PPV of treeWAS, indicating a shift from an average of zero to one false positive findings with increasing recombination. No further PPV losses are observed, however, even in S9 Appendix where frequent gene gain and loss typical of the accessory genome is simulated. Ultimately, as the F1 score in Fig 3D demonstrates, the approach adopted by treeWAS not only produces good overall performance, but by accounting for recombination, it is able to maintain good performance across a range of backgrounds, from purely clonal to frequently recombining.

### Comparison with other GWAS methods

Having described the performance of treeWAS on simulated data, we now compare it with the performance of alternative methods. Fig 2A reveals that only treeWAS and the conservative GC approach consistently rejected all false positive findings. PCA, DAPC, and the CMH test reduced FPR below the level incurred with no correction for population structure, but still returned undesirably high volumes of false positives. Fig 2A thus suggests that even the most popular dimension-reduction and cluster-based methods do not sufficiently correct for population structure in microbial contexts.

All of the non-phylogenetic approaches to correcting for population structure inherently simplify the extensive genetic relationships between isolates. The k-means clusters used in the CMH test, and the principal components of PCA and DAPC have been previously shown to correspond to the major clades and genealogical divisions of a phylogenetic tree [25, 28, 53]. In PCA, DAPC, and the CMH test, therefore, the user must make a conceptual delineation, at a given height on this tree, between what will and will not be considered parts of the population structure. Our approach, by contrast, works directly with the whole phylogenetic tree, retaining the information it provides at all levels of the clonal population structure. In addition, because the distribution of the phenotype is observed along the tips of the phylogeny but less clear in clusters and PCs, treeWAS is able to account directly for the degree of correlation observed between population structuring alleles and phenotypic clusters. The design of treeWAS therefore allows it to determine what degree of association is unlikely to have arisen by chance, given the evolutionary history inferred. For these reasons, our phylogenetic approach provides a more natural and complete solution to the problem posed by population structure, which drives the FPR gap in Fig 2A between treeWAS and its competitors.

Building on the foundation of low FPR, treeWAS is able to enhance power by drawing on the cumulative findings of multiple tests of association. The non-phylogenetic alternatives, by contrast, face an inherent trade-off between sensitivity and specificity. Fig 2B and 2C show that, consequently, none of these approaches offered simultaneously a high PPV and high sensitivity. The only non-phylogenetic method with an acceptably high PPV was GC (Fig 2C), but it also had the lowest sensitivity (Fig 2B) meaning that in our simulations it almost never found any association, correct or incorrect. PCA and DAPC, despite being too permissive of false positives, achieved only moderate sensitivities, considerably lower than treeWAS (Fig 2B). These methods undermine sensitivity by discounting higher-order lineage effects and proceed with the assumption that remaining genetic variation is ancestrally homogenous [28, 54]. Because these methods reduce power by eliminating variation with every additional PC, increasing the number of selected PCs in an effort to lower FPR in this study resulted in a complete loss of sensitivity. Our results suggest that when PCA and DAPC are used in microbial GWAS, depending on the population structure and the effect size of associations, a satisfactory trade-off between sensitivity and specificity may be unattainable.

The CMH test more effectively managed the sensitivity-specificity trade-off by applying a more conservative stratified test of association to the genetic data matrix without regressing out any relevant information. In fact, we see from Fig 2B that the sensitivity of the CMH test was only slightly lower than that of treeWAS. The CMH test also had better PPV than PCA and DAPC (Fig 2C), although it fell well below that of treeWAS. Indeed, with an mean PPV of 0.56, almost half of the results identified by the CMH test were false positives, whereas the PPV of treeWAS indicates that 92% of our findings were correct. Overall, the F1 score (Fig 2D) of CMH was similar to that of the lowest-performing individual association test within treeWAS. Yet, the CMH test achieves high F1 score values by adopting the less stringent approach of favouring high sensitivity over high PPV. In practice, it may be preferable to incur modest sensitivity losses so that the number of false positive findings may be kept at a minimum.

Additionally, Fig 3B and 3C show that because the CMH test is naive to recombination, its behaviour differed markedly from that of treeWAS. In response to increasing recombination, the CMH test maintained relatively stable sensitivity, but experienced a decrease in PPV as the number of false positive findings increased, demonstrating a lack of control for recombination. Furthermore, although the composite F1 scores in Fig 3D appear to narrow with increasing recombination, it is important to note the practical implications of the trade-offs being made by treeWAS and the CMH test. Even at the highest recombination rate examined in Fig 3, the CMH test identified only one more true positive than treeWAS. On the other hand, treeWAS found less than one false positive on average, while the CMH test found as many false positives as true positives (Fig 3C). Increasing recombination in other simulations caused the F1 score gap between the CMH test and treeWAS to increase or remain unchanged (S4 and S5 appendices).

Overall, the comparison of methods in Figs 2 and 3 indicates that the performance of the non-phylogenetic methods was limited by multiple factors: the focus on higher level population structure, the inability to control for its confounding effects in sufficient detail, the necessary trade-off between sensitivity and PPV, and the poor response to varying rates of recombination. By deliberately avoiding all of these pitfalls, the design of treeWAS achieved stronger performance on these simulated datasets.

### Application to *Neisseria meningitidis*: Identifying penicillin resistance factors

To determine whether our approach could confirm previously-identified associations, and illustrate its applicability to both binary and continuous phenotypes, we applied treeWAS to a dataset of *N. meningitidis* isolates with a penicillin resistance phenotype. We used the *Neisseria* Bacterial Isolate Genome Sequence Database (BIGSdb accessible at https://pubmlst.org/neisseria/, [55]) to download 171 *N. meningitidis* sequences from serogroup B (see S1 File), extracting both the core SNPs (166,848 SNPs) and an accessory gene presence or absence matrix (2,808 genes). We reconstructed the phylogenetic tree from whole-genome sequences with ClonalFrameML to account for recombination [42]. *N. meningitidis* has a high recombination rate, though recombination in *N. meningitidis* is not so rampant as to entirely obscure the clonal genealogy, as would be the case for example in *Helicobacter pylori* [56]. Because treeWAS accounts explicitly for the confounding effects of recombination, our approach was appropriate for this context, provided recombination-aware phylogenetic methods were used [57]. treeWAS completed the analysis of the accessory genome in 23 seconds and analysed the SNPs matrix in 24 minutes.

The penicillin resistance phenotype was analysed in two ways: as a binary and as a continuous variable. The binary phenotype was categorised according to the penicillin minimum inhibitory concentration (MIC), defining susceptible as MIC ≤ 0.06 and resistant as MIC > 0.06. The continuous phenotype was defined as the ranks of the MIC values, rather than the MIC values themselves, whose distribution was highly skewed and which were less informative than the relative MIC values.

Analysis of the accessory gene presence or absence data did not result in the identification of any gene significantly associated with either the binary or continuous penicillin resistance phenotype. However, application of treeWAS to the set of core SNPs led to the identification of many significant loci. Analysis of the binary penicillin resistance phenotype resulted in the identification of 162 significant SNPs, all of which were located in the well-characterised NEIS1753 (*penA*) gene, encoding penicillin-binding protein 2 (see S2 File and S1 Fig). This finding is consistent with the literature, which indicates that penicillin resistance in *N. meningitidis* occurs when altered forms of a penicillin-binding protein (PBP) are produced [58]. Previous work also indicates that the resistance phenotype, and the mosaic structure of *penA*, arise via homologous recombination [59, 60]. It is therefore natural that our analysis uncovered significant associations among SNPs in this gene rather than in other core or accessory genes. Indeed, the alignment displayed in S1A Fig is consistent with previous accounts in the literature describing uniformity in *penA* sequences among susceptible isolates [61] and considerable diversity among those in resistant isolates [62].

Analysis of the continuous penicillin MIC phenotype returned 30 significant SNPs (see S3 File and S2 Fig). The majority of these were also located in the *penA* gene, although SNPs were also identified in three additional genes. In the presence of antibiotics, many loci not essential to the resistance phenotype may confer a slight selective advantage [12]. For example the UDP-N-acetylmuramoylalanyl-D-glutamate–2, 6-diaminopimelate ligase is involved in cell wall formation via peptidoglycan synthesis, the process targeted by penicillin [60]. The two additional genes in which significant SNPs were identified have roles in stress response and DNA damage repair, which may not be directly related to penicillin resistance but which may instead confer a minor fitness advantage that would slightly increase MIC values. We recommend that future laboratory analyses be undertaken to determine whether the statistically significant associations between MIC and these novel candidate loci represent true causal links, via the proposed mechanisms or otherwise. It should be noted that although the classification scheme we adopted designated MIC > 0.06 as “resistant”, these isolates would in fact usually be classified as of “intermediate resistance”, as they did not exceed the standard resistance threshold of MIC > 1 (see S1 File). In light of the narrow range of MIC values in the sample, it is remarkable that treeWAS was nonetheless able to identify significant associations. By analysing resistance as a continuous variable, our approach was able to retain all of the phenotypic information available. Hence, in spite of the relatively small phenotypic effect observed, treeWAS not only retained the power to detect the central PBP gene, *penA*, but it gained sensitivity to significant SNPs in three additional genes.

### Application to *Neisseria meningitidis*: Identifying drivers of invasive disease

The overall design of treeWAS, in particular the implementation of the three association scores in Eqs 1–3, was developed with the aim of detecting genetic loci associated with subtle and complex phenotypes which may not be entirely determined by genetic factors [18]. To illustrate this, we applied treeWAS to a separate *N. meningitidis* dataset, with the more challenging phenotype of invasive disease versus carriage. Invasiveness is determined more probabilistically than penicillin resistance, on the basis of both pathogen genetics and external factors, such as host immunity [63].

From the *Neisseria* BIGSdb database, we downloaded 129 European *N. meningitidis* sequences from serogroup C (see S4 File), including both core SNPs (115,386 SNPs) and accessory gene presence or absence data (2,809 genes). ClonalFrameML was used to reconstruct the phylogenetic tree from whole-genome sequences while accounting for recombination [42]. Analyses by treeWAS of the accessory genome and core SNPs datasets were completed in 17 seconds and 7 minutes, respectively.

**Fig 4:**
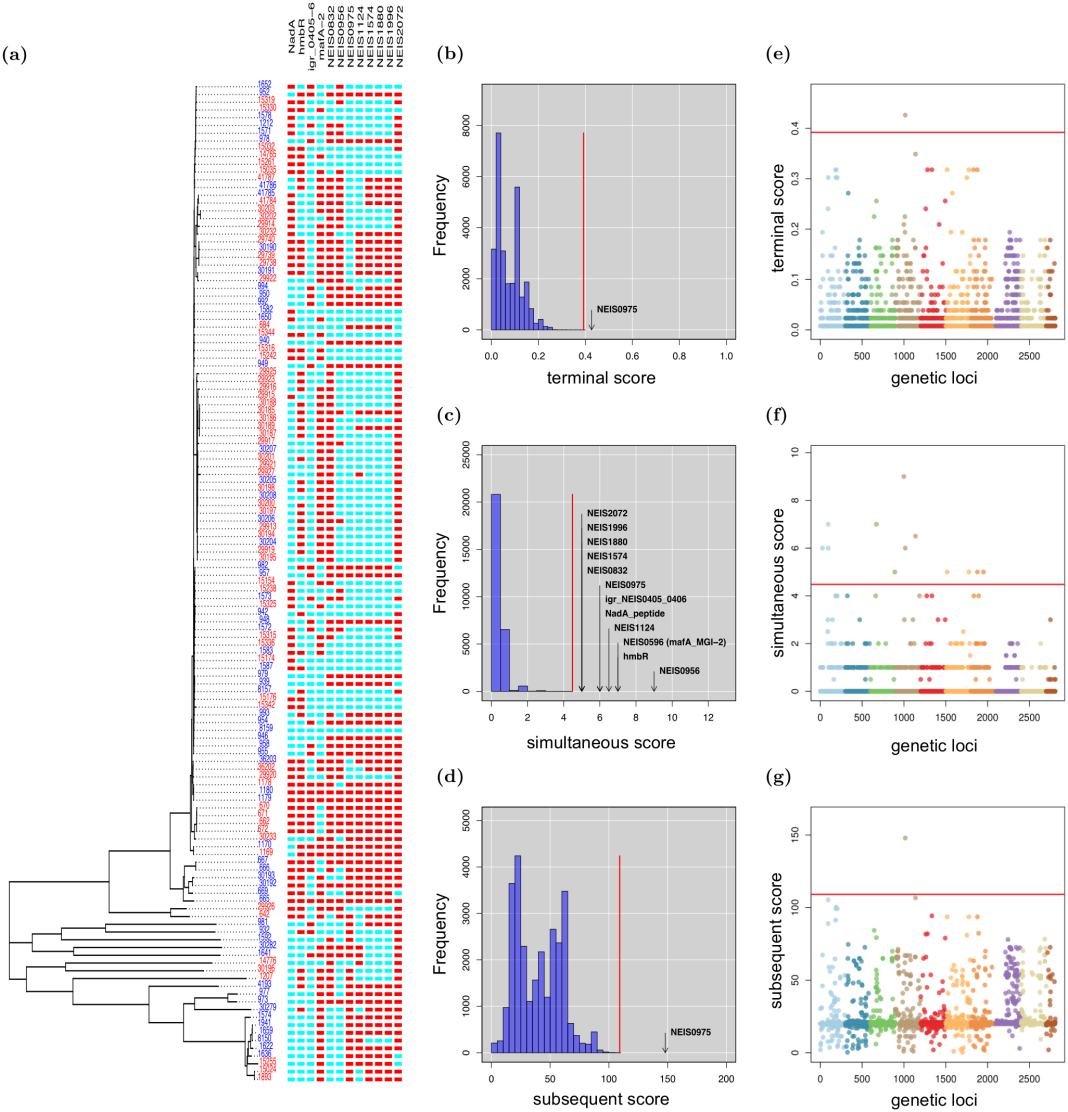
Invasive disease in the *N. meningitidis* accessory genome. treeWAS identified 12 genes associated with invasive disease. **A:** At left, the clonal genealogy reconstructed with ClonalFrameML, and terminal phenotype (blue = carrier, red = invasive). At right, an alignment of the 12 significant genes (blue = gene absence, red = gene presence). **B-D:** Null distributions of simulated association scores for (B) Score 1, (C) Score 2, (D) Score 3, a significance threshold (red), above which real associated genes are indicated. **E-G:** Manhattan plots for (E) Score 1, (F) Score 2, (G) Score 3 showing association score values for all genes, a significance threshold (red), above which points indicate significant associations.

In the analysis of the accessory gene presence or absence data, treeWAS identified 12 genes associated with carriage or invasiveness (Fig 4, Table 1). Three genes were found to be associated with invasive disease, and the role of each was confirmed by the literature. *NadA* (Neisserial adhesin A) has well-characterised roles in virulence, enabling adhesion, colonisation, and invasion of mucosal cells [64, 65]. *MafA2*, another adhesin, plays a similar role in pathogenic *Neisseria* [66, 67]. Epidemiological evidence and rat models have also linked the haemoglobin receptor protein, *hmbR*, to invasive disease in *N. meningitidis* [68, 69]. Moreover, as this gene is highly conserved, *hmbR* may be a good target for vaccine development [70].

**Table 1:** Genes associated with invasive disease in *N. meningitidis*. These genes were identified as significantly associated with invasive disease when treeWAS was applied to 129 accessory genome gene presence-or-absence sequences from *N. meningitidis* serogroup C.

Gene	Gene product
NadA_peptide	*NadA* peptide
hmbR	Haemoglobin receptor protein
igr_NEIS0405_0406	intergenic region between NEIS0405 and NEIS0406
NEIS0596 (mafA_MGI-2)	*MafA-2* adhesin
NEIS0832	hypothetical protein
NEIS0956	cell-surface protein
NEIS0975	hypothetical protein
NEIS1124	hypothetical protein
NEIS1574	DNA transport competence protein
NEIS1880	DNA transport competence protein
NEIS1996	DNA transport competence protein
NEIS2072	putative periplasmic protein

We also identified nine genes whose presence was associated with Neisserial carriage. These included the cell-surface protein encoded by NEIS0956 and the DNA transport competence proteins encoded by NEIS1574, NEIS1880, and NEIS1996, which enable genetic transformation [71, 72]. These genes may confer an adaptive advantage to *N. meningitidis* that enables immune evasion via surface modulation, and favours colonisation and survival in the nasopharyngeal niche [73]. This relationship is not entirely clear, however, as non-pathogenic carriage remains incompletely characterised at a molecular level, despite being a fundamental element of the Neisserial life cycle [74].

**Fig 5:**
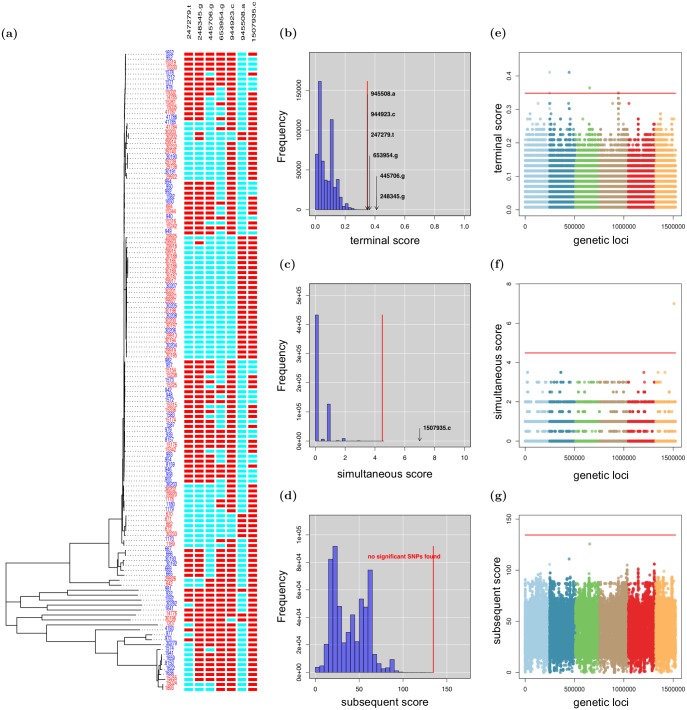
Invasive disease in *N. meningitidis* core SNPs. treeWAS identified 7 SNPs associated with invasive disease. **A:** At left, the clonal genealogy reconstructed with ClonalFrameML, and terminal phenotype (blue = carrier, red = invasive). At right, an alignment of the 7 significant SNPs (blue = allele 0; red = allele 1). **B-D:** Null distributions of simulated association scores for (B) Score 1, (C) Score 2, (D) Score 3, a significance threshold (red), above which real associated SNPs are indicated. **E-G:** Manhattan plots for (E) Score 1, (F) Score 2, (G) Score 3 showing association score values for all SNPs, a significance threshold (red), above which points indicate significant associations.

In the analysis of core SNPs, treeWAS identified seven associated loci (Fig 5, Table 2). Among these, the *porA* gene is well known for encoding a surface protein that drives hyperinvasivity in *N. meningitidis* [75–78]. Likewise, *gapA-2* may facilitate the adhesion to and invasion of host tissues [79]. As the genetic basis of invasiveness in *N. meningitidis* is not yet fully understood, we anticipate that future work will elucidate the roles of other loci in Table 2.

**Table 2:** SNPs associated with invasive disease in *N. meningitidis*. These SNPs were identified as significantly associated with invasive disease when treeWAS was applied to 129 whole-genome sequences from *N. meningitidis* serogroup C.

Locus	Gene	Gene product
247279.t	NEIS0343	N-acetylglutamate synthase
248345.g	NEIS0344	hypothetical protein
445706.g	NEIS0614	DNA ligase
653954.g	NEIS0361	hypothetical protein
934483.c	NEIS1348	hypothetical protein
945508.a	NEIS1364 (*porA*)	*PorA*, porin, class 1 outer membrane protein
1507935.c	NEIS2137 (*gapA2*)	glyceraldehyde 3-phosphate dehydrogenase C

Overall, treeWAS was able to identify both previously-known and putatively novel genes and SNPs in significant association with the commensal or invasive phenotype in *N. meningitidis*. Subsequent analyses in the laboratory would be required to confirm that a true biological or causal relationship accompanies this statistical significance.

### Conclusions

Microbial GWAS has the potential to reveal many important features of microbial genomes. Application has however been so far hampered by a lack of well founded and thoroughly tested methodology. Here we proposed a new phylogenetic approach to microbial GWAS that is able to control for the disruptive effects of both population structure and recombination, whilst still retaining a high statistical power to detect real associations. Application to both simulated and real datasets demonstrated that our method is accurate, efficient and versatile, being able to detect associations in both the core and pan-genome and for both categorical and continuous phenotypic measurements. We have implemented our approach in a user-friendly R package, treeWAS, which is freely available for public use at https://github.com/caitiecollins/treeWAS.

## Supporting information

S1 Appendix Computational time to run treeWAS. (PDF)

S2 Appendix Choosing the Method of Reconstruction. (PDF)

S3 Appendix Derivation of Score 3. (PDF)

S4 Appendix Simulation Set A (simple association). (PDF)

S5 Appendix Simulation Set B (complementary pathways). (PDF)

S6 Appendix Simulation Set C (main set). (PDF)

S7 Appendix Simulating homoplasy distributions by recombination rate with SimBac. (PDF)

S8 Appendix Significant PCs and clusters in PCA, DAPC, and CMH. (PDF)

S9 Appendix Simulation Set C (variable size). (PDF)

S1 Fig Application to penicillin resistance in *N. meningitidis* core SNPs. treeWAS identified 140 SNPs associated with penicillin resistance. **A:** At left, the clonal genealogy reconstructed with ClonalFrameML, and terminal phenotype (blue = susceptible; red = resistant). At right, an alignment of the 67 unique SNPs column patterns (blue = allele 0; red = allele 1) that were observed among the 140 significant SNPs. **B-D:** Null distributions of simulated association scores for (B) Score 1, (C) Score 2, (D) Score 3, a significance threshold (red), above which real associated SNPs are indicated. **E-G:** Manhattan plots for (E) Score 1, (F) Score 2, (G) Score 3 showing association score values for all SNPs, a significance threshold (red), above which points indicate significant associations.
(PDF)

S2 Fig Application to penicillin MIC in *N. meningitidis* core SNPs. treeWAS identified 30 SNPs associated with the ranked penicillin MIC values. **A:** At left, the clonal genealogy reconstructed with ClonalFrameML, and terminal phenotype (continuous: blue = lowest, yellow = moderate, red = highest MIC ranks). At right, an alignment of the 30 significant SNPs (blue = allele 0; red = allele 1). **B-D:** Null distributions of simulated association scores for (B) Score 1, (C) Score 2, (D) Score 3, a significance threshold (red), above which real associated SNPs are indicated. **E-G:** Manhattan plots for (E) Score 1, (F) Score 2, (G) Score 3 showing association score values for all SNPs, a significance threshold (red), above which points indicate significant associations.
(PDF)

S1 File Metadata for *N. meningitidis* isolates analysed for associations with penicillin resistance and MIC. (XLS)

S2 File Loci associated with penicillin MIC in *N. meningitidis*. These loci were identified as significantly associated with the continuous ranked penicillin MIC phenotype, when treeWAS was applied to a dataset of 171 core SNPs extracted from serogroup B *Neisseria meningitidis* whole-genome sequences.
(XLS)

S3 File Metadata for *N. meningitidis* isolates analysed for associations with invasive disease. (XLS)

S4 File Loci associated with penicillin resistance in *N. meningitidis*. These loci were identified as significantly associated with the binary penicillin resistance phenotype, resistant (MIC < = 0.06) *versus* susceptible (MIC > 0.06), when treeWAS was applied to a dataset of 171 core SNPs extracted from serogroup B *Neisseria meningitidis* whole-genome sequences.
(XLS)
