# Supplementary material for: A phylogenetic method to perform genome-wide association studies in microbes that accounts for population structure and recombination
Source: PLoS Comput Biol. 2018 Feb 5;14(2):e1005958. doi: 10.1371/journal.pcbi.1005958 (PMC5814097; doi:10.1371/journal.pcbi.1005958)
Supplement: S1 Appendix — (PDF) [file pcbi.1005958.s001.pdf]

S1 Appendix. Computational time to run treeWAS.

In this Appendix we explore the time required to run treeWAS according to dataset size. We generated 200 simulated test datasets with varying numbers of individuals and numbers of genetic loci. treeWAS was run with default arguments on each of these datasets on a standard computer and the time to completion was recorded. Time is shown in seconds, each red point represents one dataset, and a line of best fit is included in blue.

Firstly we measured the treeWAS run time for 100 datasets with numbers of individuals varying from 50 to 200 while the number of genetic loci was fixed at 10,000:

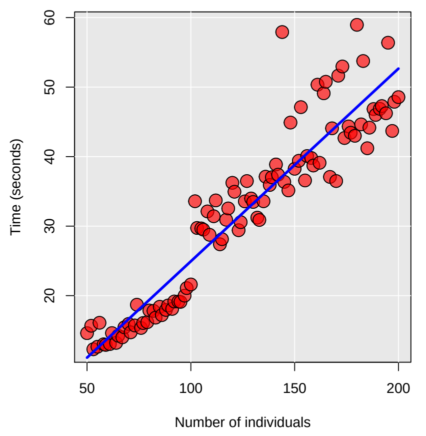

Secondly we measured the treeWAS run time for 100 datasets with numbers of genetic loci varying from 10,000 to 200,000 while the number of individuals was fixed at 100:

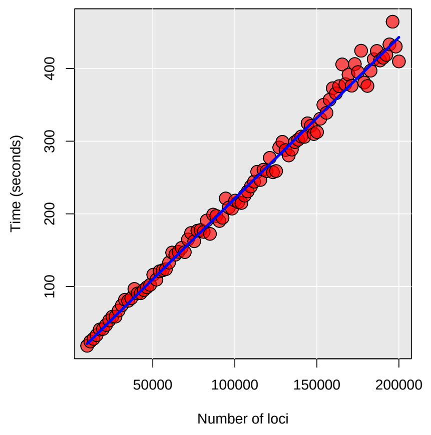

The results above show that the time required to run treeWAS is approximately linear in the number of individuals and in the number of loci considered in the GWAS study.
