# Supplementary material for: A phylogenetic method to perform genome-wide association studies in microbes that accounts for population structure and recombination
Source: PLoS Comput Biol. 2018 Feb 5;14(2):e1005958. doi: 10.1371/journal.pcbi.1005958 (PMC5814097; doi:10.1371/journal.pcbi.1005958)
Supplement: S2 Appendix — (PDF) [file pcbi.1005958.s002.pdf]

S2 Appendix. Choosing the Method of Reconstruction.

Two methods of reconstructing the ancestral states were considered: parsimony and maximum likelihood (ML). To determine whether the performance of treeWAS was affected by the method of ancestral state reconstruction, we repeated the analysis of the simulated datasets tested in this paper (Set C, $N = 80$) using ML reconstruction. As with the initial analyses using parsimony, the ML reconstruction method was used to infer the states of both the genotype and phenotype at all internal nodes. It should be noted that users are permitted to select either method of reconstruction and that the genotypic and phenotypic methods need not match. The only constraint in practice is that non-binary phenotypes must undergo an ML reconstruction.

To determine objectively which method of reconstruction gave the best results, we applied a two-sample Wilcoxon rank sum test to matched pairs of our four performance statistics (F1 score, PPV, sensitivity, FPR). We investigated a two-sided alternative hypothesis: H_A = the difference in performance between results generated with parsimony and those generated via ML was not equal to zero.

In the table below, we present a summary of the results of the Wilcoxon test. The performance of Score 1 is not affected by the method of reconstruction because it is not calculated at internal nodes. Hence, we include results for the performance of treeWAS overall as well as Scores 2 and 3 only. Adjacent to the columns containing the association score and performance statistic, the tables below include p-values for the Wilcoxon test, and then present the median difference between performance with parsimony and ML, in between the lower and upper limits of the 95% confidence interval (C.I.).

Wilcoxon test: reconstruction method

				Δ (Parsimony – ML)		
	**Association Test**	**Statistic**	**P-value**	**C.I.$_{Lower}$**	**Median**	**C.I.$_{Upper}$**
1	simultaneous	F1.score	0.0675	-0.1541	-0.0783	0.0064
2	simultaneous	PPV	0.1151	-0.2374	-0.0950	0.0354
3	simultaneous	sensitivity	0.0634	-0.1999	-0.1000	0.0000
4	simultaneous	FPR	0.3045	0.0000	0.0001	0.0001
5	subsequent	F1.score	0.0516	0.0000	0.1056	0.2137
6	subsequent	PPV	0.6089	-0.2251	0.0417	0.3590
7	subsequent	sensitivity	0.0656	-0.0001	0.1000	0.2000
8	subsequent	FPR	0.6598	-0.0001	0.0000	0.0001
9	treeWAS.all	F1.score	0.4652	-0.0952	-0.0292	0.0555
10	treeWAS.all	PPV	0.2416	-0.1454	-0.0514	0.0416
11	treeWAS.all	sensitivity	0.2403	-0.1500	-0.0500	0.0499
12	treeWAS.all	FPR	0.2522	0.0000	0.0000	0.0001

The results of this Wilcoxon test include no p-values below 0.05 and, hence, show that the performance of treeWAS is not significantly affected by the method of ancestral state reconstruction.

This finding left us at liberty to select a reconstruction method for treeWAS to use by default and within the analyses included in this paper. We selected parsimony over ML as the former fits more closely with the association testing paradigm adopted in

treeWAS. As with parsimony, both Scores 2 and 3 that rely on this reconstruction exclude branch length from their calculation. As simultaneous substitutions may indicate association whether they occur on long or short branches, Score 2 measures association without reference to branch length. Likewise, as the S1 Appendix shows, the performance of Score 3 is improved by ignoring branch length. Hence, because parsimony similarly overlooks branch lengths while ML does not, we selected a parsimonious reconstruction.

Nevertheless, the evidence suggests that both parsimony and ML produce similar levels of performance in treeWAS. As such, both reconstruction methods are made available within treeWAS for users to explore.
