# Supplementary material for: A phylogenetic method to perform genome-wide association studies in microbes that accounts for population structure and recombination
Source: PLoS Comput Biol. 2018 Feb 5;14(2):e1005958. doi: 10.1371/journal.pcbi.1005958 (PMC5814097; doi:10.1371/journal.pcbi.1005958)
Supplement: S3 Appendix — (PDF) [file pcbi.1005958.s003.pdf]

S3 Appendix. Derivation of Score 3.

The correlation score, C_x, can be described in terms of two components, P_x and G_x, representing, respectively, the probability of the value of the phenotype and genotype at a point x on a branch of length l. These probabilities are identified using a linear interpolation between the known or reconstructed states at the ancestral and descendant nodes of the branch.

$$P_x \; = \frac{1}{l}(p_i^{anc}(l-x) \; + \; p_i^{des}x) \tag{1}$$

$$G_x = \frac{1}{l}(g_i^{anc}(l-x) \; + \; g_i^{des}x) \tag{2}$$

C_x represents the degree of association between phenotype and genotype at point x.

$$C_x \; = P_xG_x + (1-P_x)(1-G_x) - P_x(1-G_x) - (1-P_x)G_x \tag{3}$$

Expanding C_x to be described in terms of its component parts in full, we get:

$$\begin{aligned} C_x \; =& \frac{p_i^{anc}(l-x) \; + \; p_i^{des}x}{l}\frac{g_i^{anc}(l-x) \; + \; g_i^{des}x}{l} + \\ & \left(1-\frac{p_i^{anc}(l-x) \; + \; p_i^{des}x}{l}\right)\frac{g_i^{anc}(l-x) \; + \; g_i^{des}x}{l} - \\ & \frac{p_i^{anc}(l-x) \; + \; p_i^{des}x}{l}\left(1-\frac{g_i^{anc}(l-x) \; + \; g_i^{des}x}{l}\right) - \\ & \left(1-\frac{p_i^{anc}(l-x) \; + \; p_i^{des}x}{l}\right)\frac{g_i^{anc}(l-x) \; + \; g_i^{des}x}{l} \end{aligned} \tag{4}$$

Score 3 is defined as the absolute sum for all branches i of the integral of C_x, when the point x takes positions along the branch i, that is, when x is between 0 and l_i:

$$\textbf{Score 3} = |\sum_{i=1}^{n_b} \int_0^l C_x dx \; | \tag{5}$$

The integral can be solved mathematically, resulting in the following equation:

$$\begin{aligned} \textbf{Score 3} = |\sum_{i=1}^{n_b} \; & (-1+2p_i^{anc})(-1+2g_i^{anc})l_i \; - \\ & (-p_i^{anc} + p_i^{des} - g_i^{anc} + 4p_i^{anc}g_i^{anc} - \\ & 2p_i^{des}g_i^{anc} + g_i^{des} - 2p_i^{anc}g_i^{des})l_i \; + \\ & \frac{4}{3}(p_i^{anc} - p_i^{des})(g_i^{anc} - g_i^{des})l_i \; | \end{aligned} \tag{6}$$

Simplifying this results in the final equation for Score 3, which is calculated across all branches of the tree and for each variable site:

$$\begin{aligned} \textbf{Score 3} = |\sum_{i=1}^{n_b} \; & l\frac{4}{3}p_i^{anc}g_i^{anc} \; + \; \frac{2}{3}p_i^{anc}g_i^{des} \; + \; \frac{2}{3}p_i^{des}g_i^{anc} \; + \; \frac{4}{3}p_i^{des}g_i^{des} \\ & - \; p_i^{anc} \; - \; p_i^{des} \; - \; g_i^{anc} \; - \; g_i^{des} \; + \; 1 \; | \end{aligned} \tag{7}$$

Experimental validation on simulated data indicates that the performance of Score 3 is improved by removing the branch length term, l, resulting in the final equation for Score 3, which gives all edges equal weight.

To design an optimal third score, we explored whether Score 3 would give better results with the branch length term, l_i, excluded or included. For all simulated datasets ($N = 80$), we repeated the calculation of Score 3, without branch length (Score3_{NoBL}) and with branch length (Score3_{BL}). We then ran a two-sample Wilcoxon rank sum test on matched pairs of our four performance statistics under the two conditions. The results are presented in the table below. Rows containing a significant p-value ($p < 0.05$) are highlighted in yellow.

Wilcoxon test: Score 3 branch length

			Δ (**Score3$_{\mathbf{NoBL}}$ − Score3$_{\mathbf{BL}}$**)		
	**Statistic**	**P-value**	**C.I.$_{\mathbf{Lower}}$**	**Median**	**C.I.$_{\mathbf{Upper}}$**
1	F1.score	0.0147	0.0123	0.1041	0.1628
2	PPV	0.5261	-0.3214	-0.0461	0.1917
3	sensitivity	0.0137	0.0001	0.1000	0.2000
4	FPR	0.2986	0.0000	0.0000	0.0001
