# Supplementary material for: A phylogenetic method to perform genome-wide association studies in microbes that accounts for population structure and recombination
Source: PLoS Comput Biol. 2018 Feb 5;14(2):e1005958. doi: 10.1371/journal.pcbi.1005958 (PMC5814097; doi:10.1371/journal.pcbi.1005958)
Supplement: S4 Appendix — (PDF) [file pcbi.1005958.s004.pdf]

S4 Appendix. Simulation Set A (simple association).

In addition to the more complex simulation of associations analysed within the paper (Set C), a simpler simulation procedure was also explored (Set A). Apart from the simulation of the phenotype and associated loci, all other parameters remain identical to those in Set C.

In Set A, associations between the ten associated loci and the phenotype are created in a three-step procedure:

1. The phenotype is simulated.
    (a) The expected value for the number of phenotypic substitutions to occur on the tree (e.g., 15) is provided as input and set to be the λ parameter of a Poisson distribution.
    (b) A single "true" value, N, for the number of phenotypic substitutions to occur on the tree in this round of Simulation Set A is drawn from this Poisson distribution.
    (c) These N phenotypic substitutions are assigned to the branches of the tree with probability proportion to branch length, such that longer branches are more likely to contain phenotypic substitutions. Branches are sampled without replacement so that the number of phenotypic substitutions is equal to N (i.e., Only one substitution may be assigned to each branch to prevent negation by subsequent substitutions).
    (d) A phenotypic state for the root node is drawn at random and, accounting for the locations of the phenotypic substitutions, the pattern of phenotypic states is identified at all internal and terminal nodes.
2. Perfect association is generated.
    (a) The ten associated loci are mapped onto the tree in perfect correspondence with the phenotype, undergoing substitutions on the same branches and resulting in 100% correlation at all nodes in the tree.
3. Association is "diluted" by switching the state of the genetic variable at a subset of the terminal nodes.
    (a) A dilution factor (e.g., 10%) is used to randomly identify the specified proportion of *terminal* nodes for each associated locus.
    (b) The state of the associated locus is switched to the alternate binary state at these terminal nodes, weakening the association between the genetic variable site in question and the phenotype.
    (c) Note that with a dilution factor of 10%, correlation between the associated loci and the phenotype will be approximately 90%, and Score 1 at these loci will be approximately 0.8 (= (90 - 10)/100).

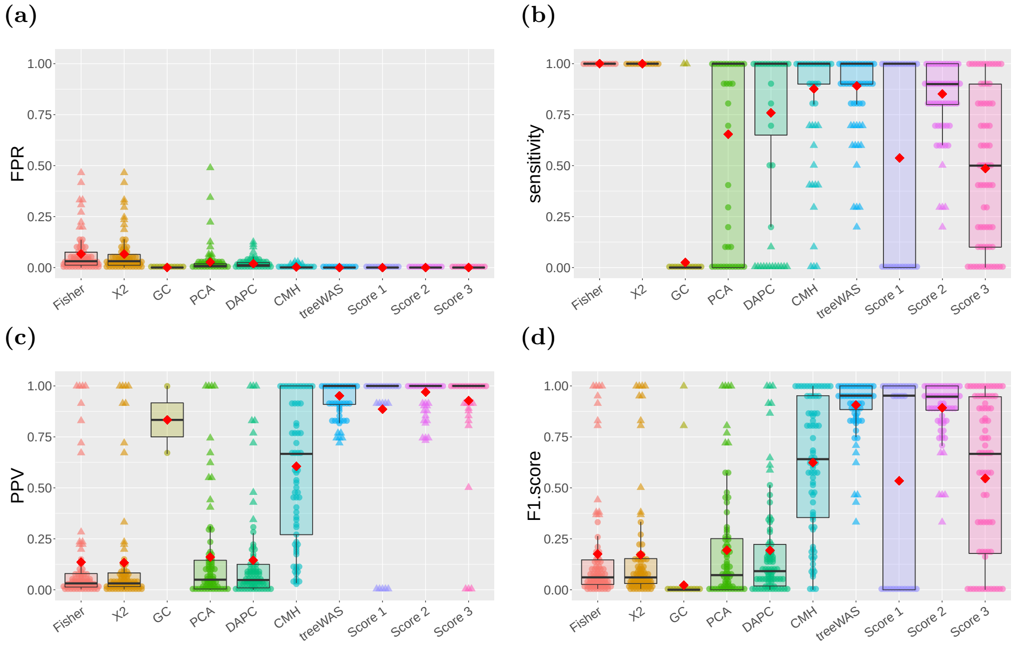

Performance by association test (Set A). The performance on simulated datasets for the six comparator GWAS methods and treeWAS, alongside its three association tests individually, is summarised along the four metrics of evaluation. This figure includes simulated datasets from all levels of recombination (N = 80).

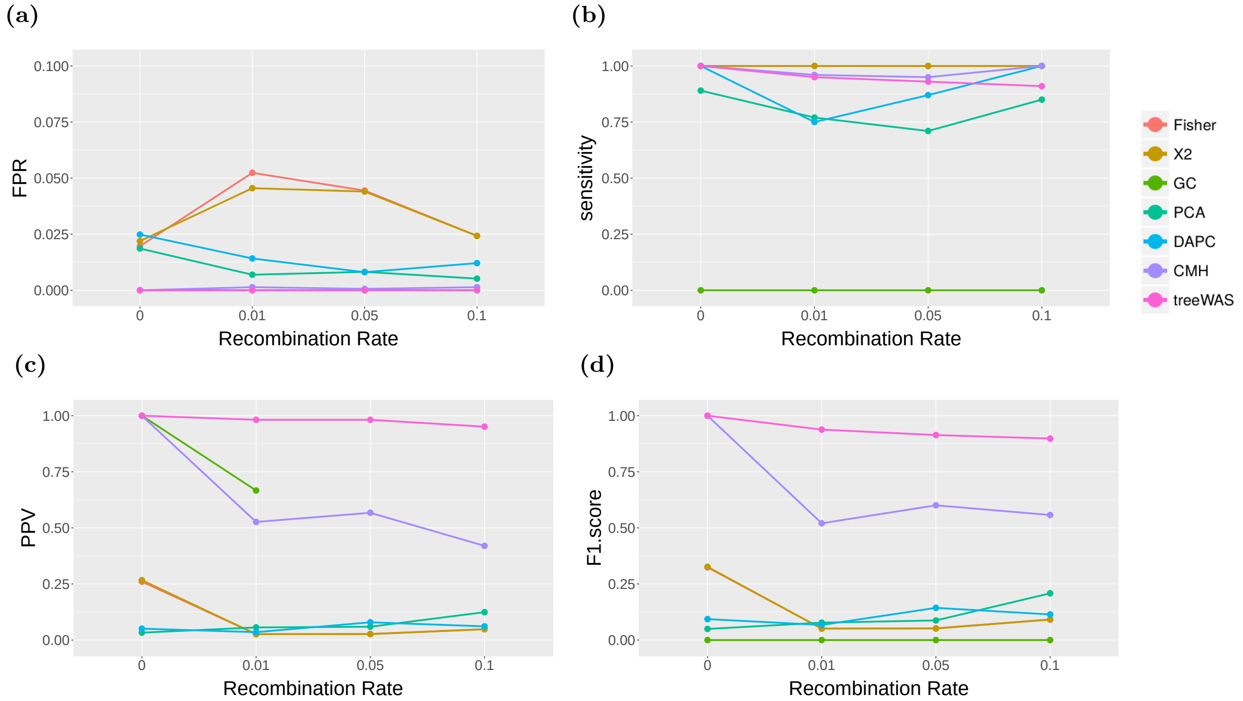

Performance by recombination rate (Set A). Interquartile mean performance by association test and recombination rate. **A:** False Positive Rate. **B:** Sensitivity. **C:** Positive Predictive Value. **D:** F1 Score.

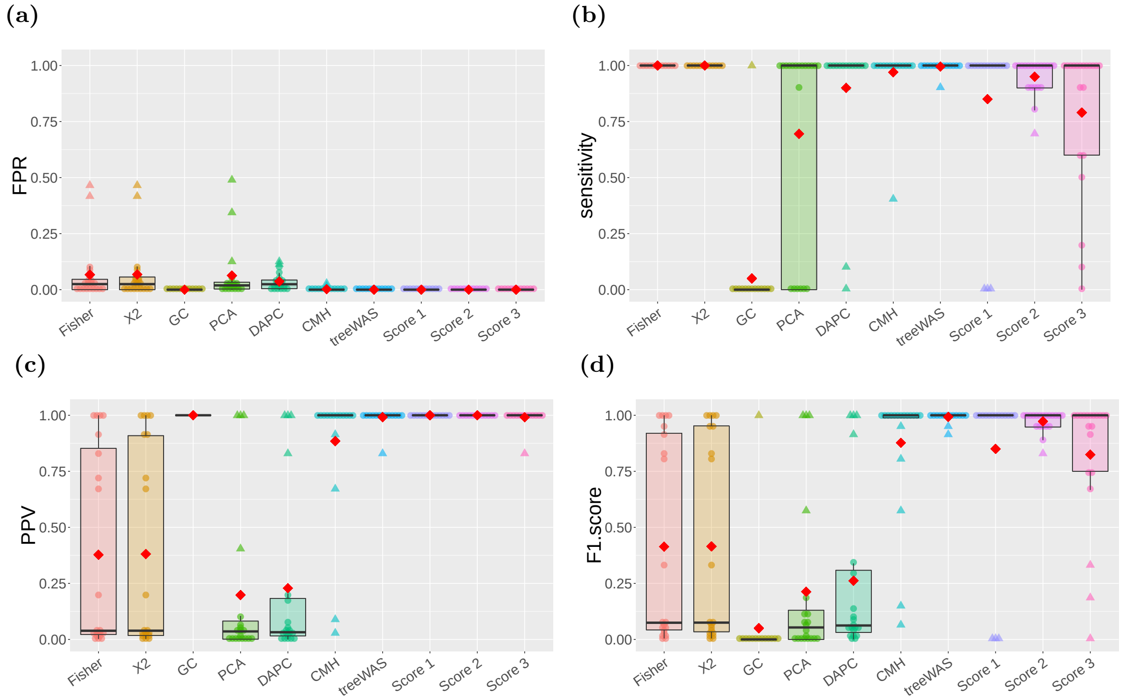

Performance by association test (Set A, R=0). This figure includes simulated datasets from Set A with no recombination (N = 20).

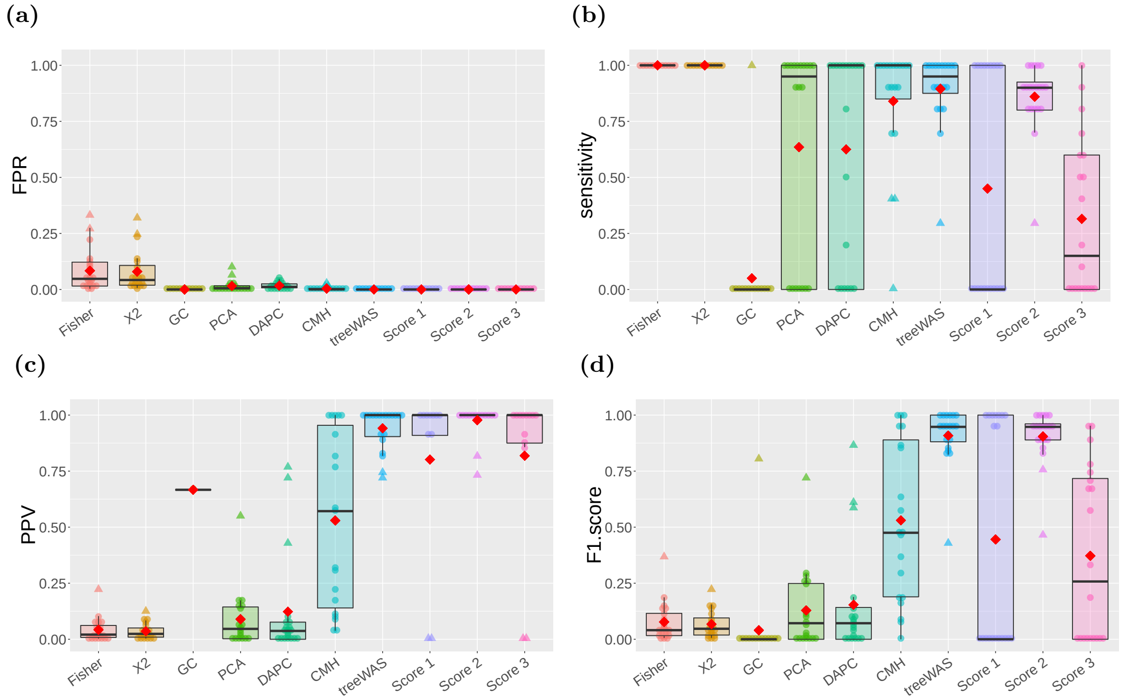

Performance by association test (Set A, R=0.01). This figure includes simulated datasets from Set A with R = 0.01 (N = 20).

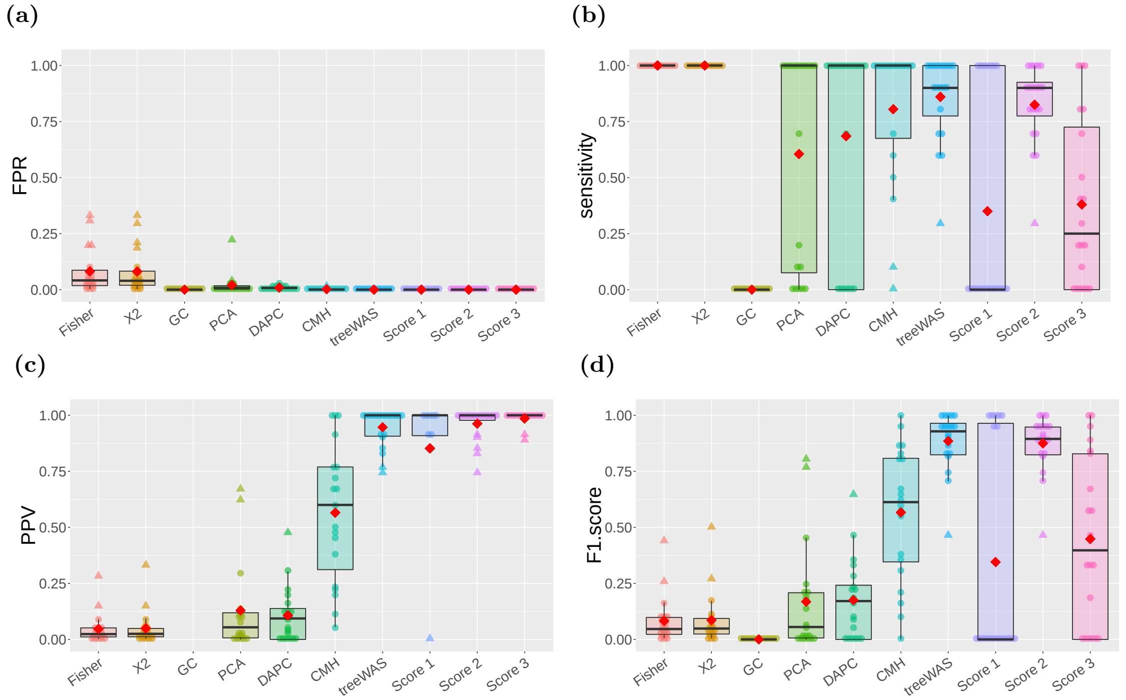

Performance by association test (Set A, R=0.05). This figure includes simulated datasets from Set A with R = 0.05 (N = 20).

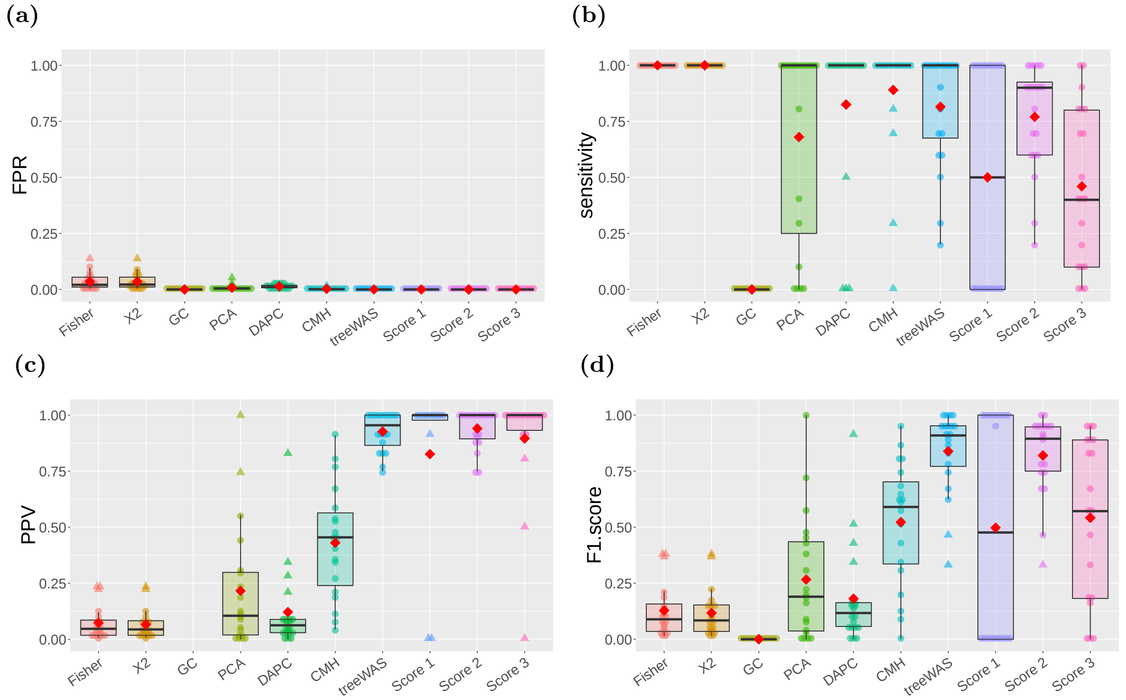

Performance by association test (Set A, R=0.1). This figure includes simulated datasets from Set A with R = 0.1 (N = 20).
