# Supplementary material for: A phylogenetic method to perform genome-wide association studies in microbes that accounts for population structure and recombination
Source: PLoS Comput Biol. 2018 Feb 5;14(2):e1005958. doi: 10.1371/journal.pcbi.1005958 (PMC5814097; doi:10.1371/journal.pcbi.1005958)
Supplement: S5 Appendix — (PDF) [file pcbi.1005958.s005.pdf]

S5 Appendix. Simulation Set B (complementary pathways).

In addition to the complex and simple methods of simulating associations performed in Sets C and A, we also applied all methods of GWAS to a third simulated set (Set B) to explore whether associations giving rise to the phenotype through complementary pathways could be identified. Apart from the simulation of the phenotype and associated loci, all other parameters remain identical to those in Set C.

In Set B, complementary associations between the genetic loci and the phenotype are created as follows:

1. The phenotype is simulated exactly as in Set A, Step (1).
2. Perfect association is generated, again, as in Set A, Step (2).
3. Two complementary pathways are created and associations are divided between the two.
   (a) The phylogenetic tree is divided into two major subtrees. This is accomplished by identifying major clades and then, if either clade falls below a minimum threshold of one third of the number of terminal nodes, transferring smaller clades from the larger to the smaller subtree until two sets of clades between one third and two thirds of the number of terminal nodes are identified.
   (b) To generate complementarity, the first five of ten associated loci are maintained in perfect association in one of the two subtrees, and their states are changed to 0 in the other subtree. The same pattern, in the other subtree, is generated in the second set of five associated loci.

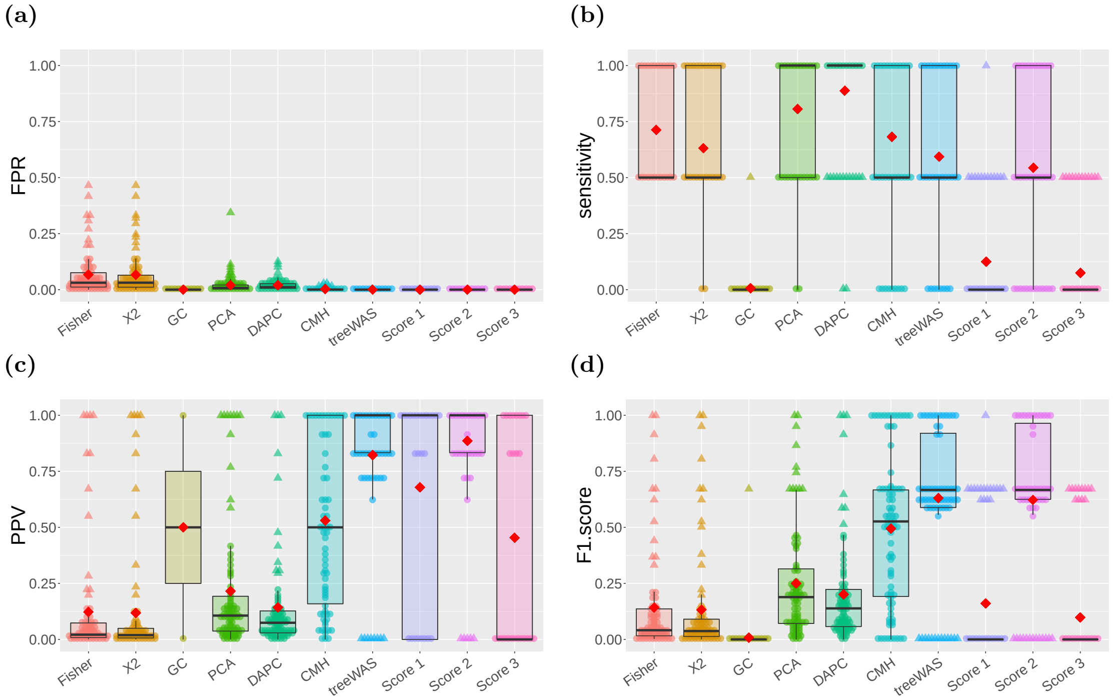

Performance by association test (Set B). The performance on simulated datasets for the six comparator GWAS methods and treeWAS, alongside its three association tests individually, is summarised along the four metrics of evaluation. This figure includes simulated datasets from all levels of recombination (N = 80).

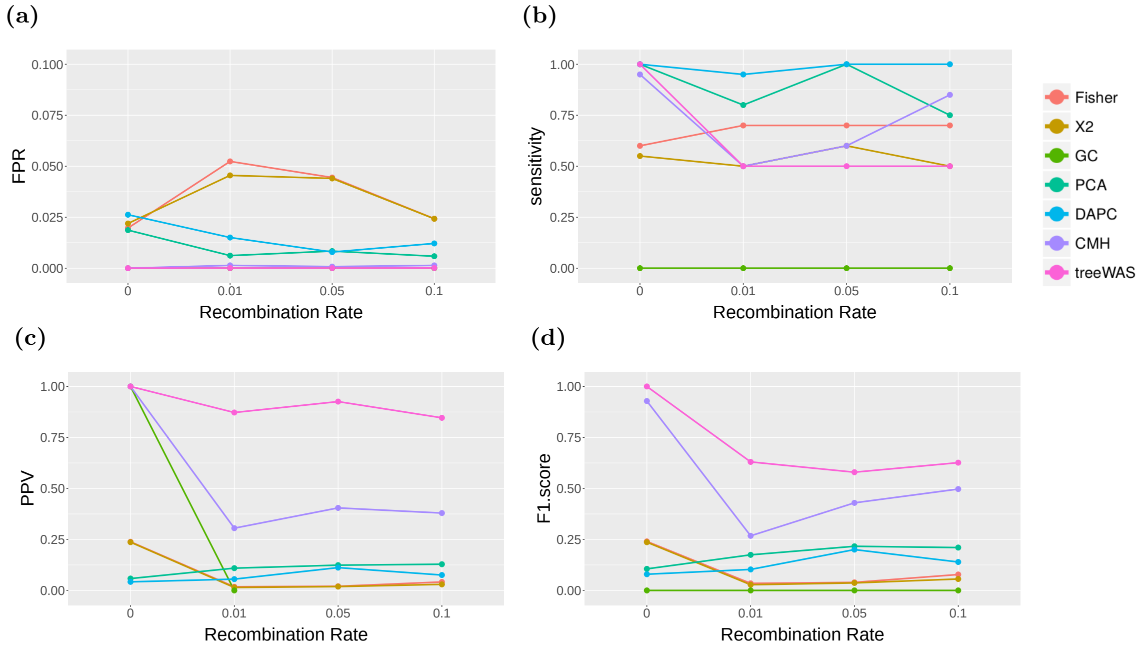

Performance by recombination rate (Set B). Interquartile mean performance by association test and recombination rate. **A:** False Positive Rate. **B:** Sensitivity. **C:** Positive Predictive Value. **D:** F1 Score.

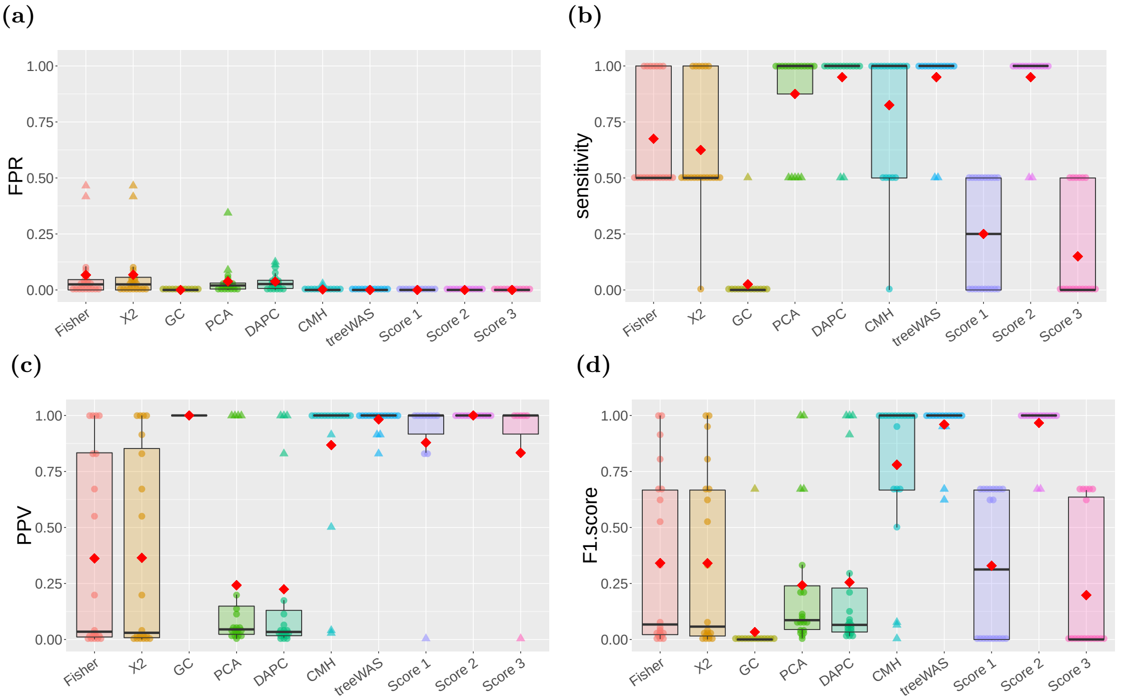

Fig 9. Performance by association test (Set B, R=0). This figure includes simulated datasets from Set B with no recombination (N = 20).

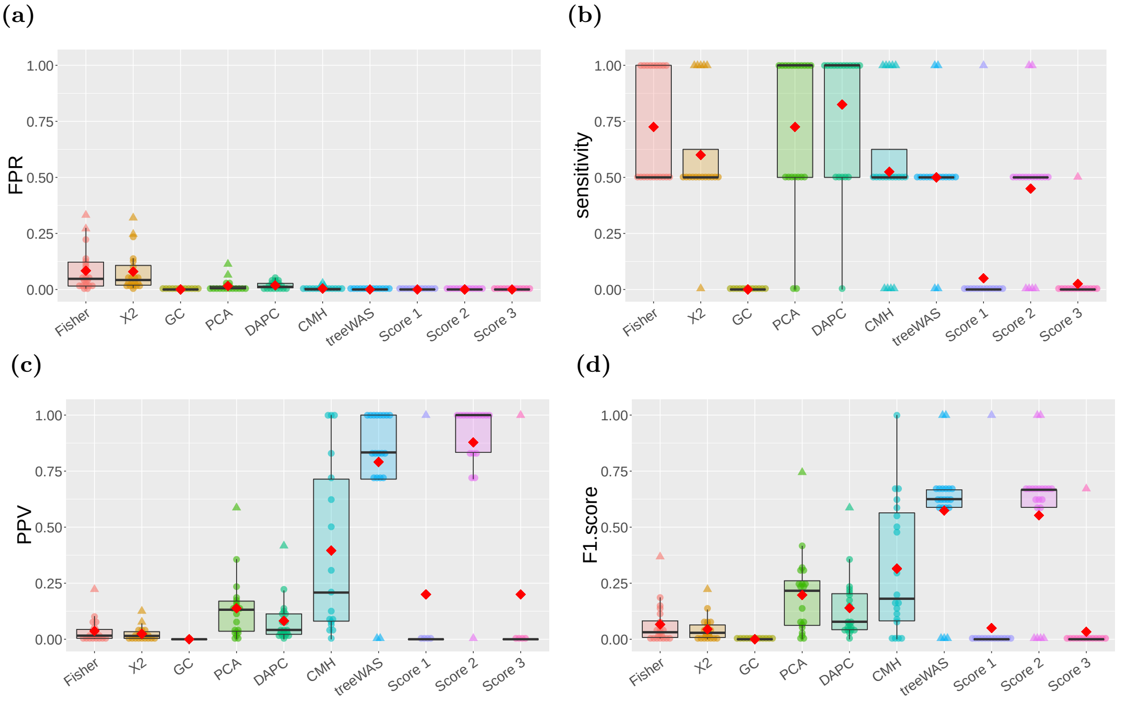

Fig 10. Performance by association test (Set B, R=0.01). This figure includes simulated datasets from Set B with R = 0.01 (N = 20).

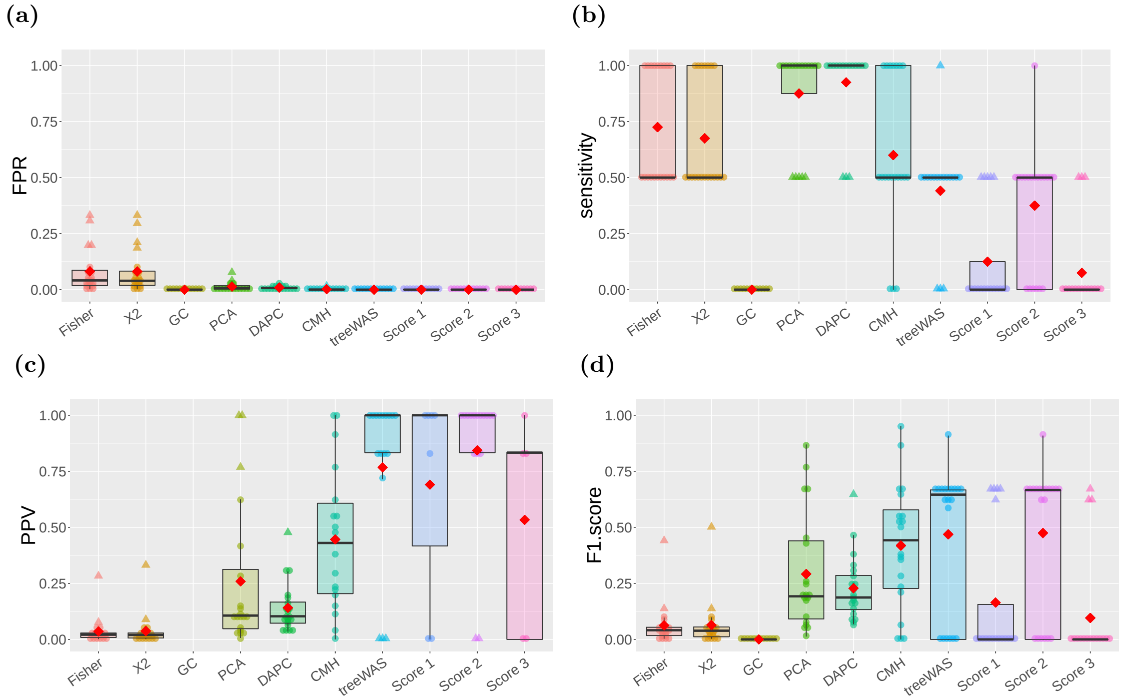

Performance by association test (Set B, R=0.05). This figure includes simulated datasets from Set B with R = 0.05 (N = 20).

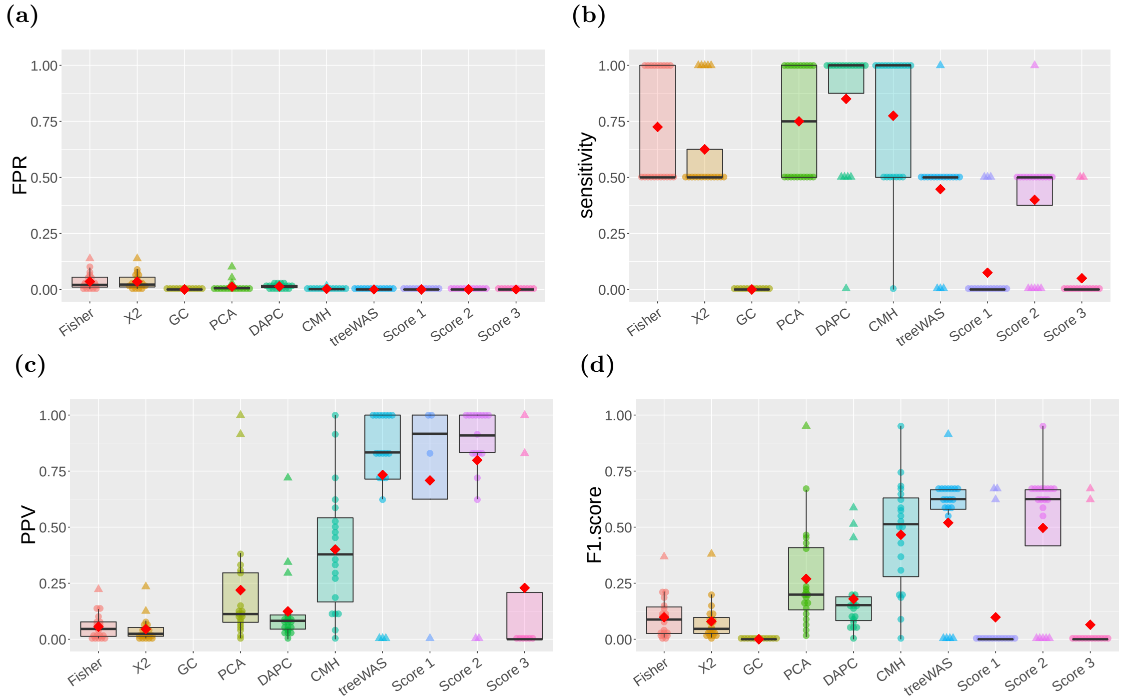

Performance by association test (Set B, R=0.1). This figure includes simulated datasets from Set B with R = 0.1 (N = 20).
