# Supplementary material for: A phylogenetic method to perform genome-wide association studies in microbes that accounts for population structure and recombination
Source: PLoS Comput Biol. 2018 Feb 5;14(2):e1005958. doi: 10.1371/journal.pcbi.1005958 (PMC5814097; doi:10.1371/journal.pcbi.1005958)
Supplement: S6 Appendix — (PDF) [file pcbi.1005958.s006.pdf]

S6 Appendix. Simulation Set C (main set).

Simulation Set C is the main set of simulations which was used in the article. The method of simulation is described in the article, as well as some of the most important results obtained with this set. This Appendix contains additional results using obtained when using a specific value of the recombination rate (R=0, 0.01, 0.05, 0.1). It also includes results on datasets with a small number of loci (emulating gene presence/absence data rather than SNPs), and results on datasets of variable sizes in terms of the number of genomes and number of SNPs.

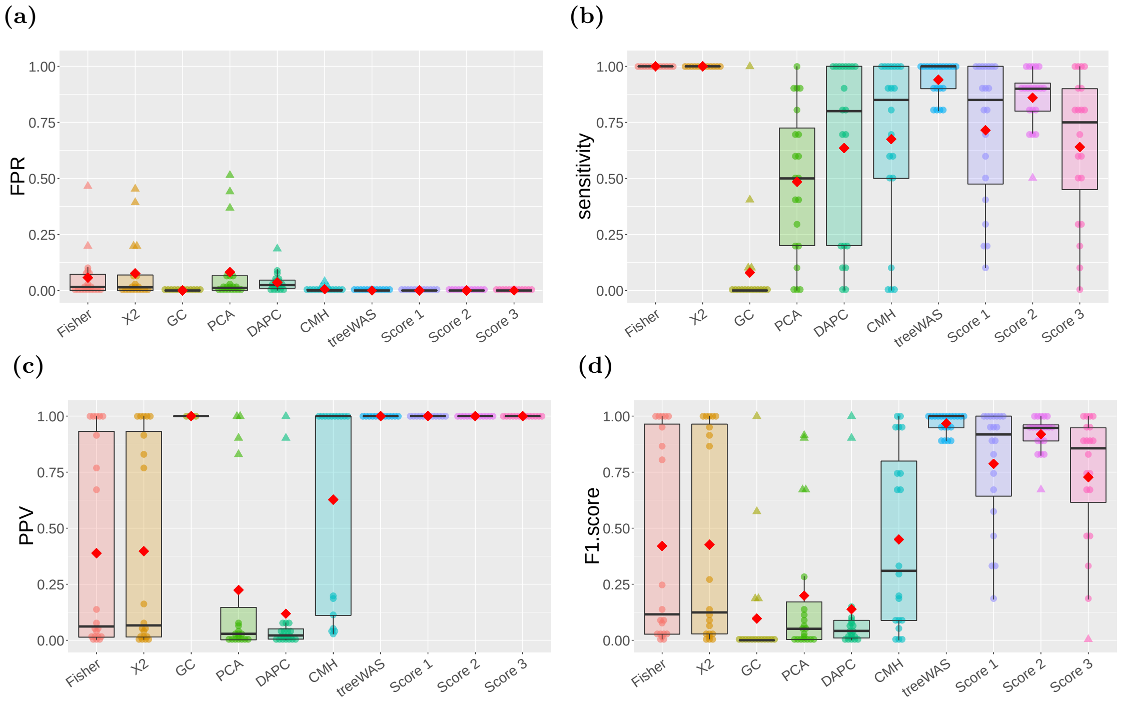

Performance by association test (Set C, R=0). This figure includes simulated datasets from Set C with no recombination (N = 20).

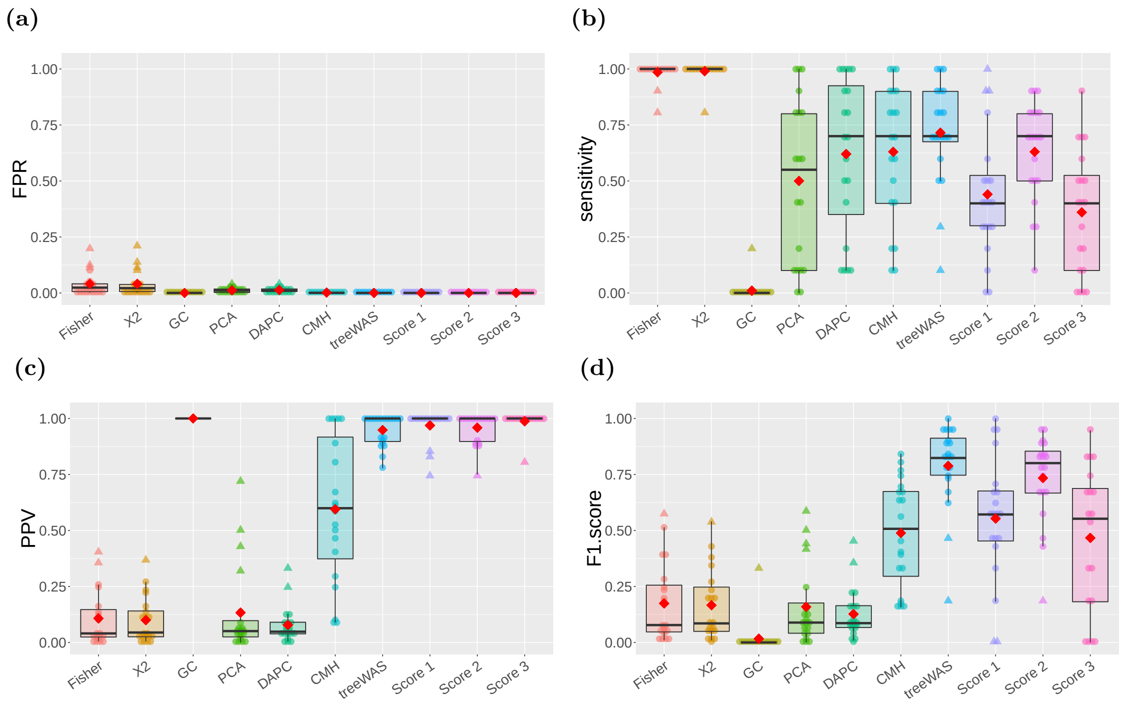

Performance by association test (Set C, R=0.01). This figure includes simulated datasets from Set C with R = 0.01 (N = 20).

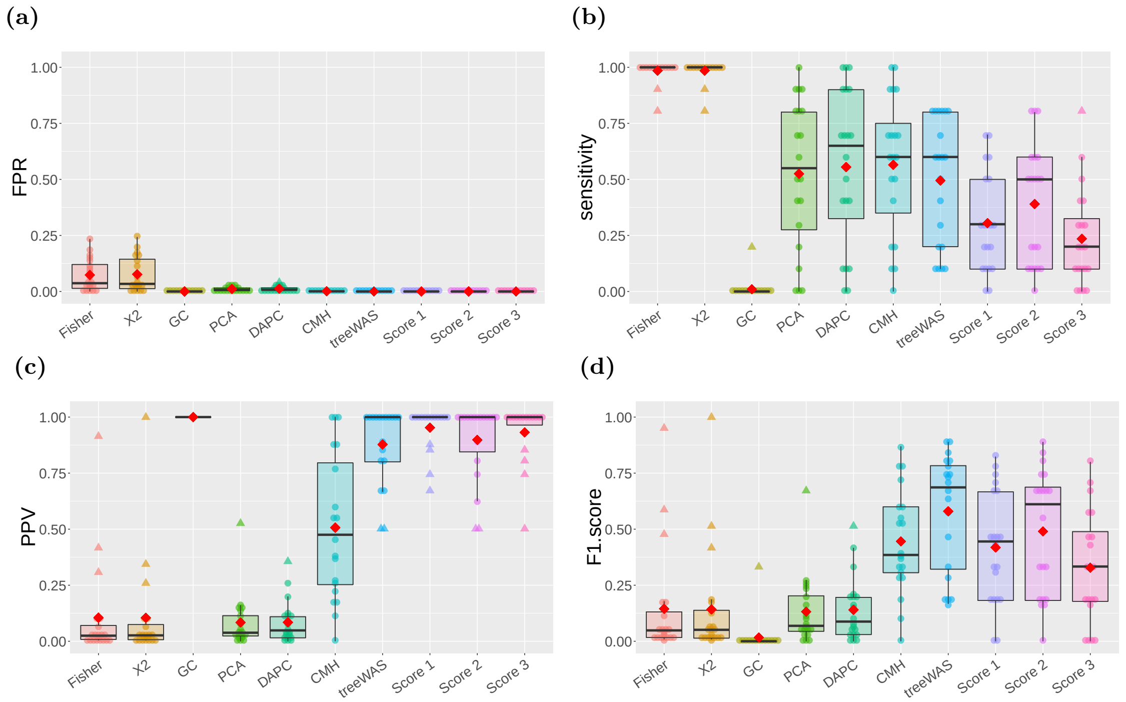

Performance by association test (Set C, R=0.05). This figure includes simulated datasets from Set C with R = 0.05 (N = 20).

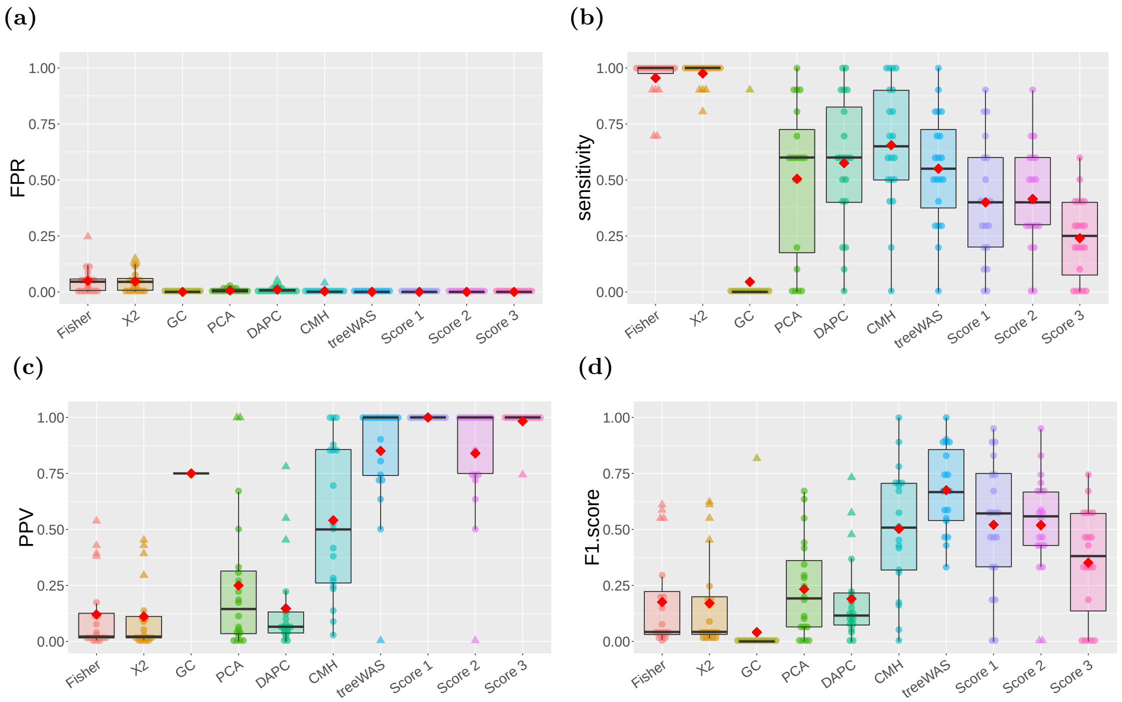

Performance by association test (Set C, R=0.1). This figure includes simulated datasets from Set C with R = 0.1 (N = 20).
