# Supplementary material for: A phylogenetic method to perform genome-wide association studies in microbes that accounts for population structure and recombination
Source: PLoS Comput Biol. 2018 Feb 5;14(2):e1005958. doi: 10.1371/journal.pcbi.1005958 (PMC5814097; doi:10.1371/journal.pcbi.1005958)
Supplement: S7 Appendix — (PDF) [file pcbi.1005958.s007.pdf]

S7 Appendix. Simulating homoplasy distributions by recombination rate with SimBac.

To generate homoplasy distributions characteristic of varying recombination rates, we used the SimBac software (Brown et al 2016, Microbial Genomics 2:10.1099/mgen.0.000044), with default parameters (-N = 100, -T = 0.01, -m = 0, -M = 0, -r = 0, -D = 500, -e = 500, -B = 10000, -G = 0), to generate genetic datasets with recombination rates, -R, of 0, 0.01, 0.05, and 0.1, and extracted the homoplasy distributions.

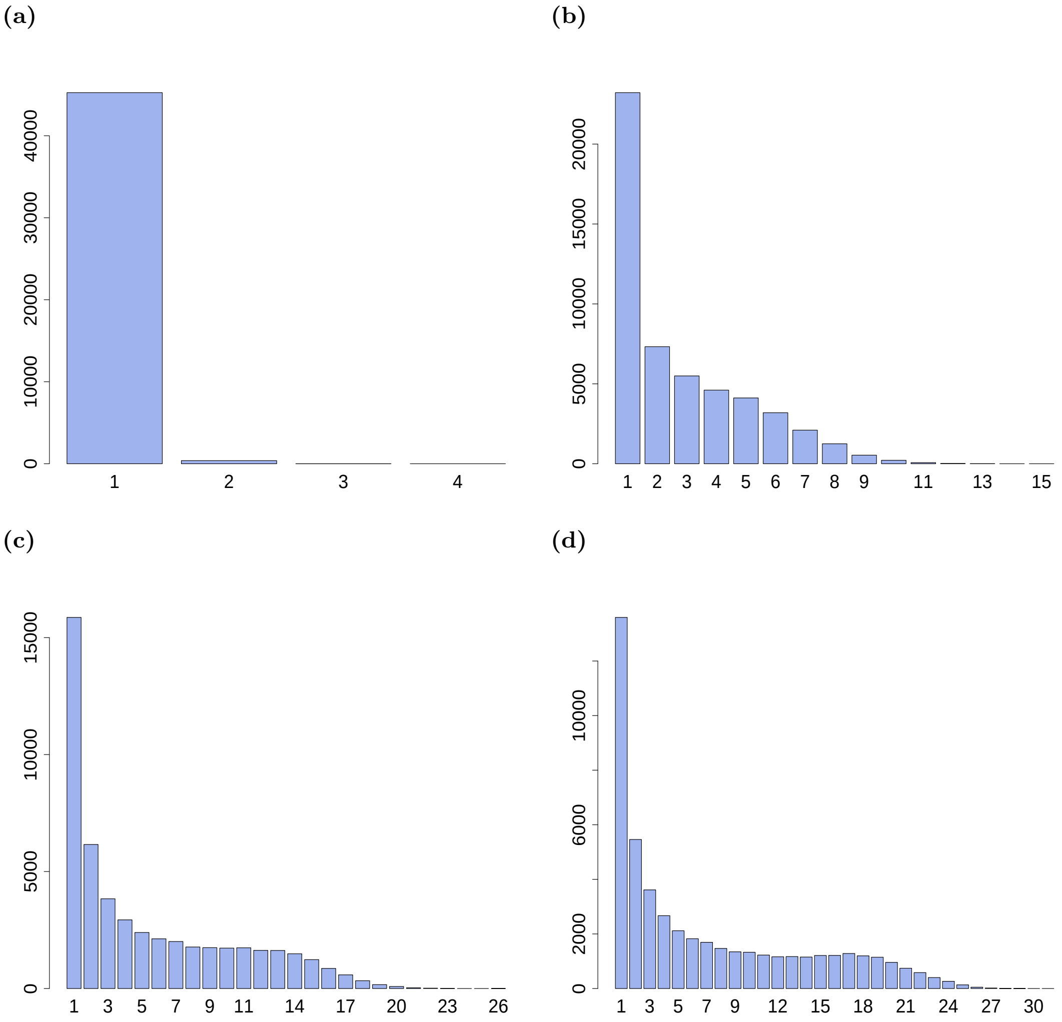

SimBac homoplasy distributions by recombination rate. Four homoplasy distributions generated with SimBac at different within-species recombination rates (R) are represented with histograms showing the number of substitutions on the x-axis and the number of loci on the y-axis. **A:** R = 0. **B:** R = 0.01. **C:** R = 0.05. **D:** R = 0.1.
