# Supplementary material for: A phylogenetic method to perform genome-wide association studies in microbes that accounts for population structure and recombination
Source: PLoS Comput Biol. 2018 Feb 5;14(2):e1005958. doi: 10.1371/journal.pcbi.1005958 (PMC5814097; doi:10.1371/journal.pcbi.1005958)
Supplement: S8 Appendix — (PDF) [file pcbi.1005958.s008.pdf]

S8 Appendix. Significant PCs and clusters in PCA, DAPC, and CMH.

The number of significant dimensions to be retained in PCA and DAPC, and the number of clusters to be retained in the CMH test were determined with the aid of k-means clustering (Lee et al 2009, BMC Bioinformatics 10:S73). By ensuring that all three of these approaches to correcting or accounting for population structure were based on the same model, we removed this source of variation from the comparative analysis. The optimal number of populations, k, to describe the population structure of a dataset was determined by applying k-means clustering to the PCA-transformed genetic data, and selecting the k associated with the lowest Bayesian Information Criterion value (Lee et al 2009, BMC Bioinformatics 10:S73).

The number of significant principal components (PCs) of PCA was set as the number of dimensions required to separate k populations, while accounting for no less than 60% of the total variance of the data. In the correction via DAPC, the number of PCs of PCA retained in the first step of the DAPC analysis was determined by stratified cross-validation. And the number of principal components (PCs) of the Discriminant Analysis step of DAPC was set as the number of dimensions required to separate k populations. Likewise, k was set as the number of clusters to be used in stratifying the analysis for the CMH test.
