# Supplementary material for: A phylogenetic method to perform genome-wide association studies in microbes that accounts for population structure and recombination
Source: PLoS Comput Biol. 2018 Feb 5;14(2):e1005958. doi: 10.1371/journal.pcbi.1005958 (PMC5814097; doi:10.1371/journal.pcbi.1005958)
Supplement: S9 Appendix — (PDF) [file pcbi.1005958.s009.pdf]

S9 Appendix. Simulation Set C (variable size).

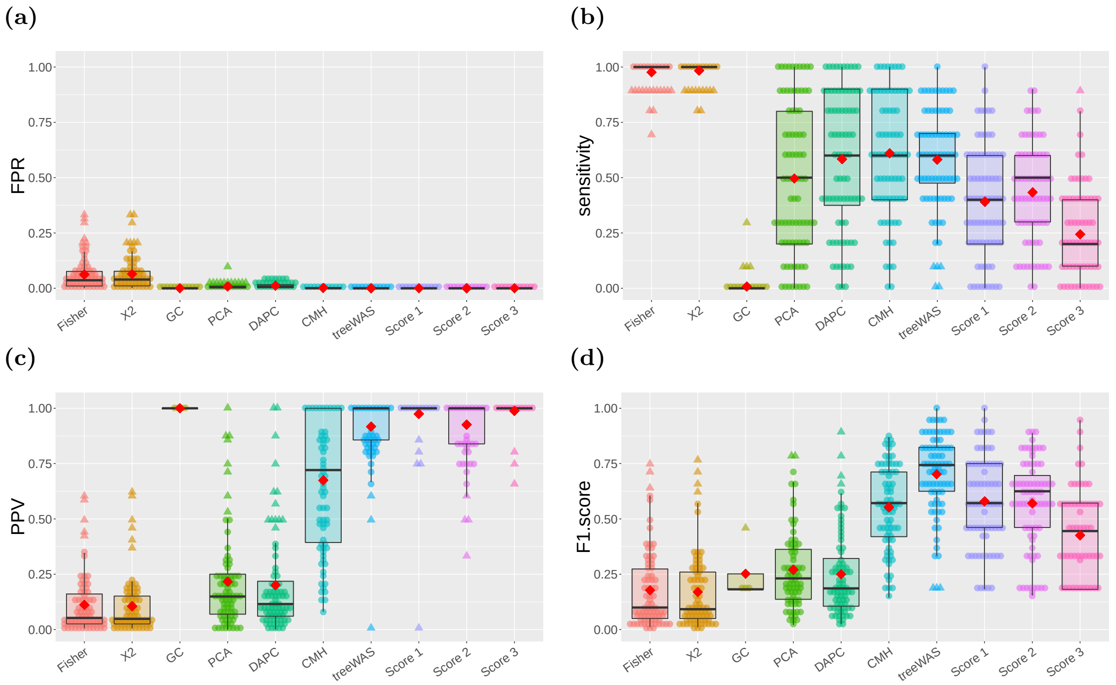

Performance by association test (Set C, accessory genome). Each association testing method was applied to a set of simulated datasets containing 100 individuals and 5,000 genetic loci, a size typical of gene presence-or-absence data (N = 80). Datasets were simulated with a relatively high level of recombination, R = 0.2, so that performance could be examined under conditions of frequent gain and loss of genetic elements.

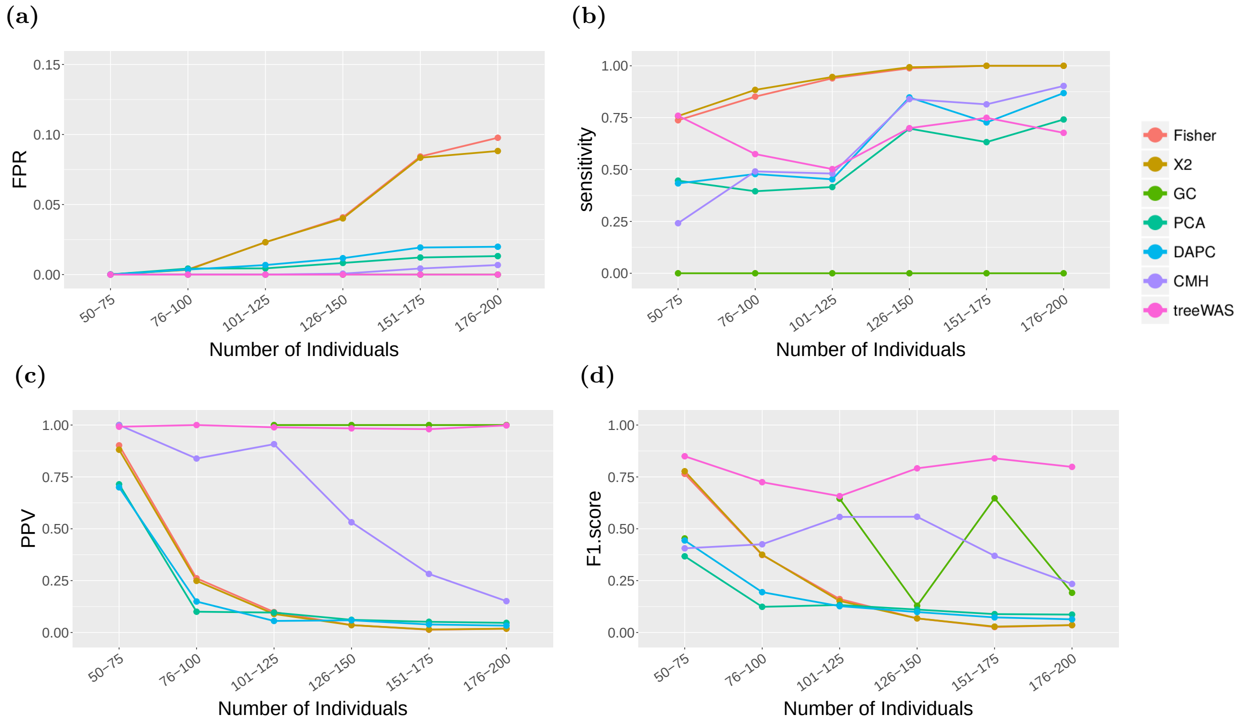

Performance by number of individuals (Set C). Each association testing method was applied to datasets simulated across a range of sizes, ranging the number of individuals from 50 to 200 and the number of loci from 10,000 to 100,000 (N = 80). All simulated datasets in this figure were generated with R = 0.01. Here, the interquartile mean performance of each association testing method is presented by number of individuals.

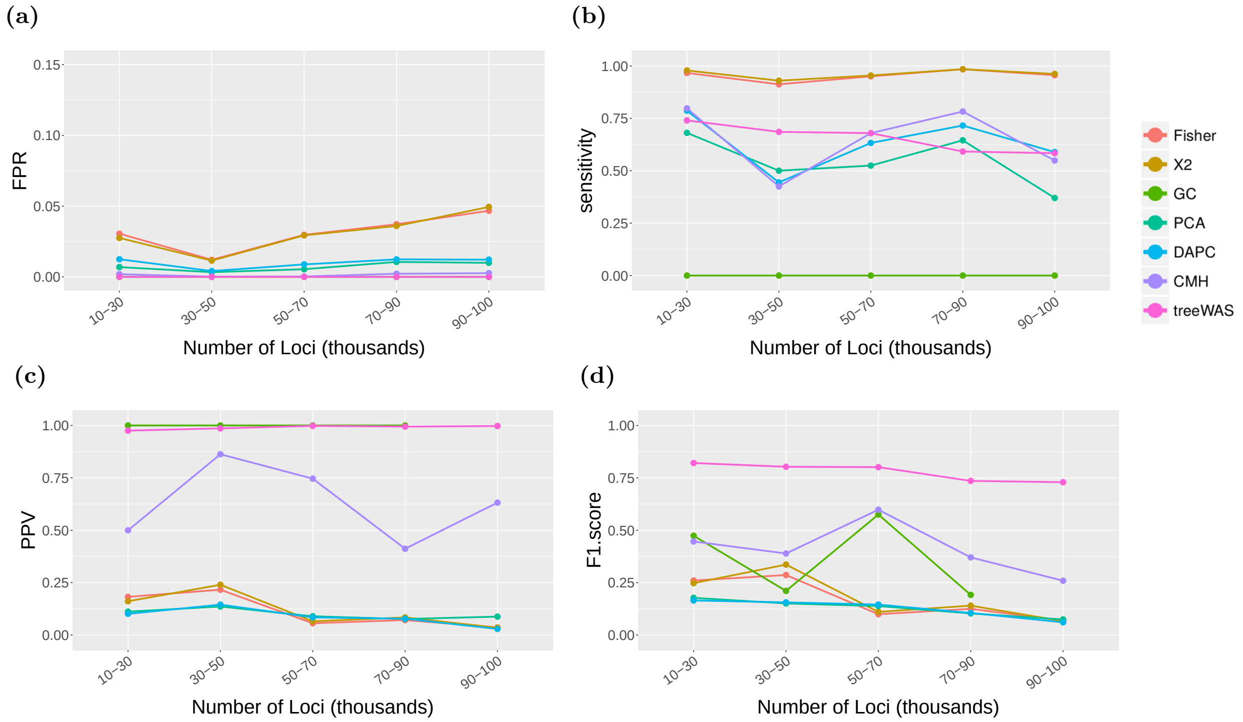

Performance by number of genetic loci (Set C). Each association testing method was applied to datasets simulated across a range of sizes, ranging the number of individuals from 50 to 200 and the number of loci from 10,000 to 100,000 (N = 80). All simulated datasets in this figure were generated with R = 0.01. Here, the interquartile mean performance of each association testing method is presented by number of genetic loci.
