# Supplementary figures and images for: A phylogenetic method to perform genome-wide association studies in microbes that accounts for population structure and recombination
Source: PLoS Comput Biol. 2018 Feb 5;14(2):e1005958. doi: 10.1371/journal.pcbi.1005958 (PMC5814097; doi:10.1371/journal.pcbi.1005958)

(a)

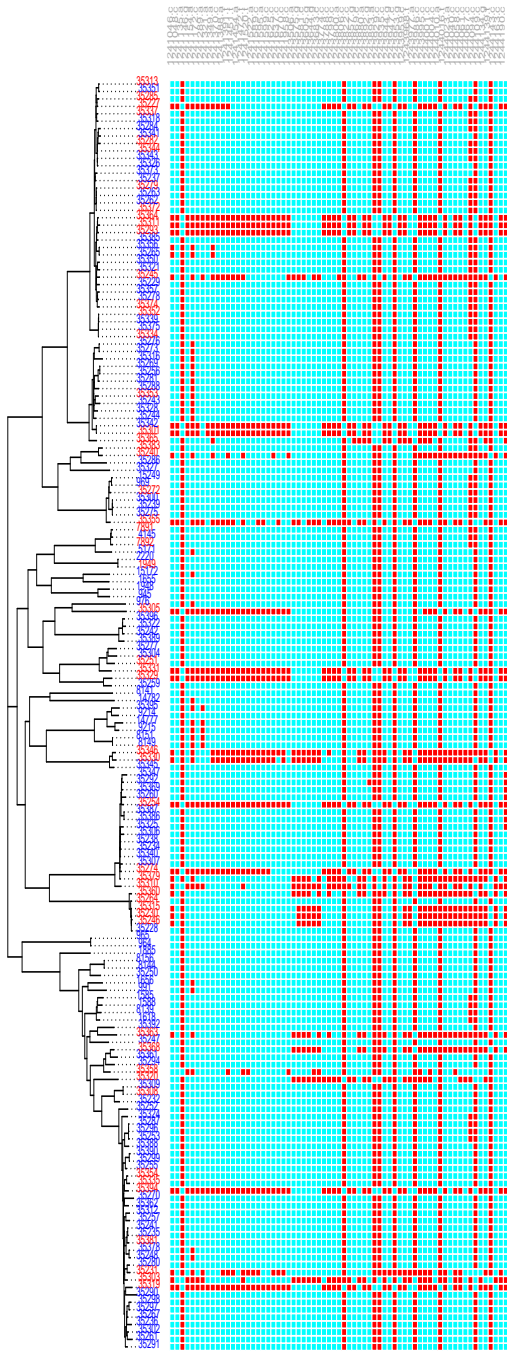

(b)

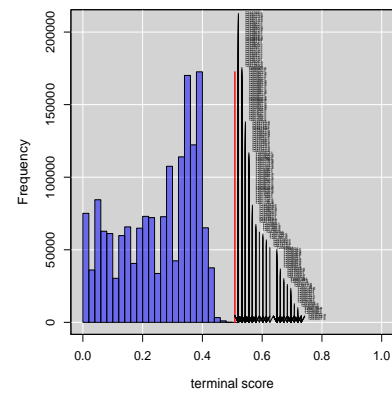

(e)

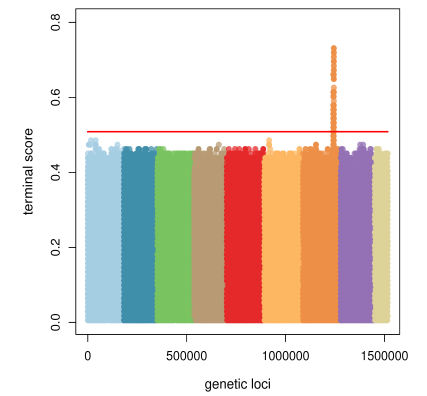

(c)

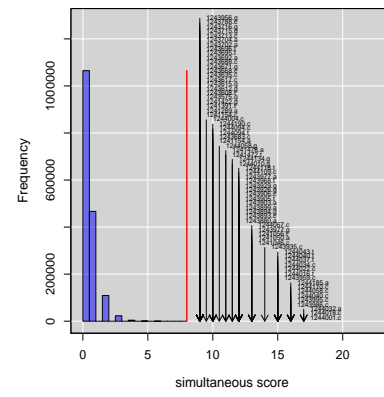

(f)

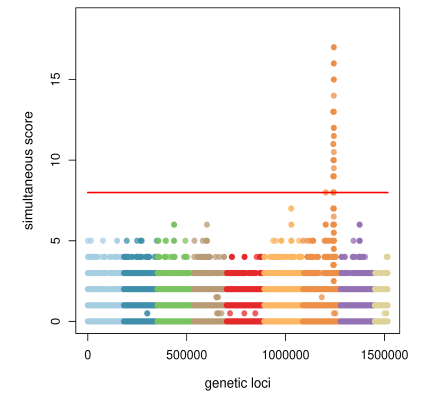

(d)

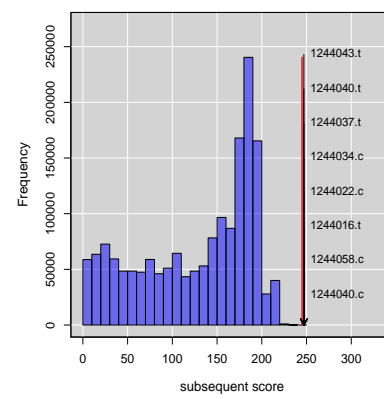

(g)

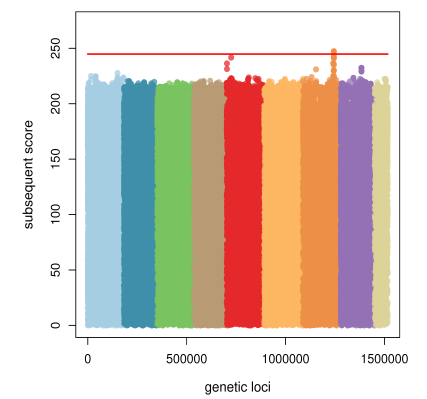

Supplement: S1 Fig — treeWAS identified 140 SNPs associated with penicillin resistance. A: At left, the clonal genealogy reconstructed with ClonalFrameML, and terminal phenotype (blue = susceptible; red = resistant). At right, an alignment of the 67 unique SNPs column patterns (blue = allele 0; red = allele 1) that were observed among the 140 significant SNPs. B-D: Null distributions of simulated association scores for (B) Score 1, (C) Score 2, (D) Score 3, a significance threshold (red), above which real associated SNPs are indicated. E-G: Manhattan plots for (E) Score 1, (F) Score 2, (G) Score 3 showing association score values for all SNPs, a significance threshold (red), above which points indicate significant associations. (PDF) [file pcbi.1005958.s010.pdf]

(a)

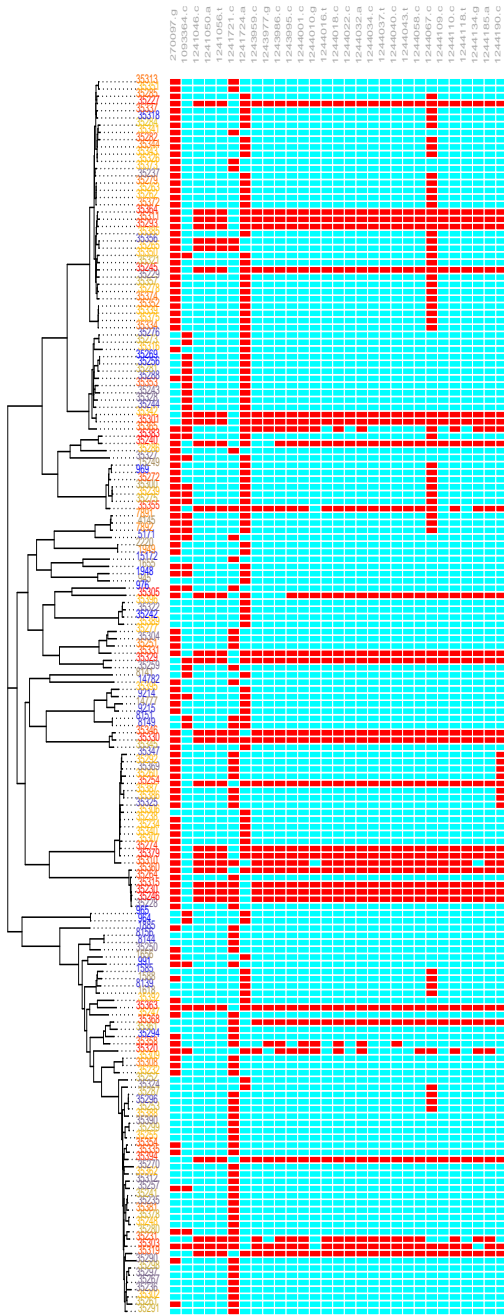

(b)

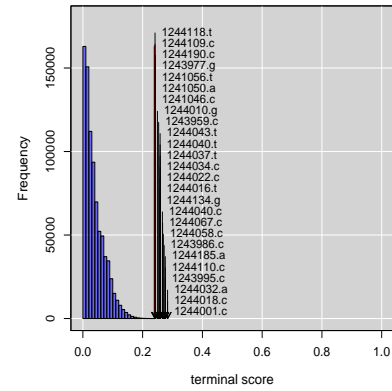

(e)

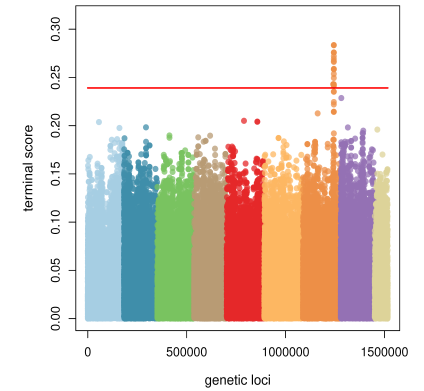

(c)

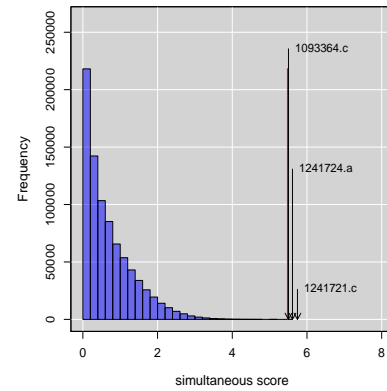

(f)

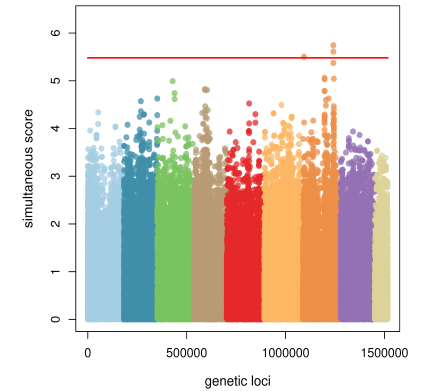

(d)

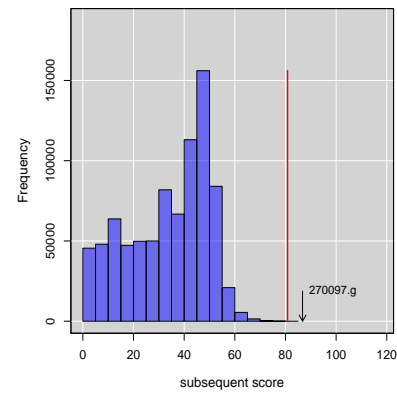

(g)

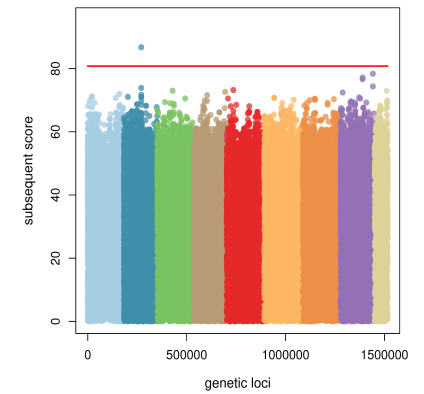

Supplement: S2 Fig — treeWAS identified 30 SNPs associated with the ranked penicillin MIC values. A: At left, the clonal genealogy reconstructed with ClonalFrameML, and terminal phenotype (continuous: blue = lowest, yellow = moderate, red = highest MIC ranks). At right, an alignment of the 30 significant SNPs (blue = allele 0; red = allele 1). B-D: Null distributions of simulated association scores for (B) Score 1, (C) Score 2, (D) Score 3, a significance threshold (red), above which real associated SNPs are indicated. E-G: Manhattan plots for (E) Score 1, (F) Score 2, (G) Score 3 showing association score values for all SNPs, a significance threshold (red), above which points indicate significant associations. (PDF) [file pcbi.1005958.s011.pdf]
